# Associations of Ultra-Processed Food Intake and Its Circulating Metabolomic Signature with Mental Disorders in Middle-Aged and Older Adults

**DOI:** 10.3390/nu17091582

**Published:** 2025-05-04

**Authors:** Shenghao Yuan, Tengfei Zhu, Jiawei Gu, Li Hua, Jinli Sun, Xiaobei Deng, Jinjun Ran

**Affiliations:** School of Public Health, Shanghai Jiao Tong University School of Medicine, Shanghai 200025, China; scp-173@sjtu.edu.cn (S.Y.); zhutengfei@shsmu.edu.cn (T.Z.); jw.gu0312@sjtu.edu.cn (J.G.); seyhuali@shsmu.edu.cn (L.H.); sunjinli1989@shsmu.edu.cn (J.S.); jinjunr@sjtu.edu.cn (J.R.)

**Keywords:** dietary factors, ultra-processed foods, metabolomic, mental disorder, psychological symptoms, mediation analysis

## Abstract

Background: The global rise in ultra-processed food (UPF) consumption and the persistent burden of mental disorders have raised growing public health concerns. Emerging evidence suggests that unfavorable dietary patterns, particularly with high UPF intake, contribute to the development of mental disorders. Objective: To assess the associations of UPF-related metabolic signatures and mental disorders. Methods: In this population-based cohort study of 30,059 participants from the UK Biobank, we first identified a plasma metabolic signature associated with UPF intake leveraging nuclear magnetic resonance metabolomics. We then applied Cox and logistic regression models to investigate the associations of both UPF consumption and its metabolic signature with incident mental disorders and specific psychological symptoms, respectively. Results: Higher UPF intake was significantly associated with increased risks of overall mental disorder (hazard ratio per 10% increment [95% confidence interval]: 1.04 [1.02, 1.06]), depressive disorder (1.14 [1.08, 1.20]), anxiety disorder (1.12 [1.06, 1.18]), and substance use disorder (1.06 [1.01, 1.11]), as well as several psychological symptoms including suicidal ideation (odds ratios [95% confidence interval]: 1.12 [1.03, 1.16]) and anxiety feeling (1.05 [1.01, 1.09]). Similarly, the UPF-related metabolic signature was independently associated with elevated risks of these mental health outcomes and partially mediated the associations between UPF intake and mental disorders. Conclusions: These findings highlighted the potential metabolic pathways underlying the neuropsychiatric risks of UPF consumption and underscored the importance of dietary quality in mental health.

## 1. Introduction

Mental disorders, encompassing conditions such as depression, anxiety, and substance use disorder, are ranked as one of the primary contributors to global disability and disease burden [1]. Despite advancements in pharmacological and psychological therapies, the global prevalence of mental disorders has remained stagnant in recent decades, underscoring the urgent need for complementary prevention strategies targeting modifiable risk factors [2]. An increasing body of research indicates that dietary habits are crucial in the development and advancement of mental disorders [3]. Specifically, diets rich in vegetables, fruits, and omega-3 fatty acids have been linked to a lower risk of mental disorders, while pro-inflammatory and Western diets high in saturated fats, sugars, and refined carbohydrates are linked to a heightened risk [4,5,6,7]. These findings indicate that modifying nutritional habits might represent an effective approach to early intervention of mental disorders [8]. Among the various dietary contributors to poor health, ultra-processed foods (UPFs) have emerged as an urgent issue due to the widespread consumption and detrimental health effects [9].

Defined by the NOVA classification, UPFs are mass-produced food items that undergo multiple processing steps and contain little whole-food content [10,11]. The consumption of UPFs has been steadily increasing worldwide, driven by urbanization and globalization of food systems [12]. Their poor nutritional profile, marked by elevated caloric content, minimal dietary fiber, and excessive amounts of saturated fats, sugars, and sodium, has been linked to an elevated risk of a range of chronic conditions such as mental disorders [13,14]. Several large-scale observational studies have found that higher UPF intake is positively associated with increased risks of anxiety and depressive disorder [15,16]. In addition, high sugar intake, one of the characteristics of UPFs, has been linked to an increased vulnerability to substance use disorders [17]. However, due to the scarcity of clinical trials and experimental studies, the current evidence remains insufficient to confirm the causal relationship.

Although substantial evidence has implicated UPFs in mental health disturbances, the pathways through which they affect the brain and nervous system remain incompletely understood. Mechanisms such as oxidative stress, hypothalamic–pituitary–adrenal (HPA) axis dysregulation, endocrine disruption, and gut microbiota alterations have been proposed and are believed to impact neurobiological processes, including neuroinflammation, synaptic plasticity, and neurotransmitter regulation [18,19]. These biological processes can be reflected by changes in circulating metabolites [20]. Therefore, metabolomics, which can systematically capture the body’s metabolic responses to UPF exposures across multiple biological pathways, provides a promising approach. However, large-scale cohort studies examining these associations using high-throughput metabolomics are still lacking. Furthermore, while psychological symptoms constitute core manifestations of mental disorders and are key to early identification, the specific link between UPF-induced metabolic shifts and clinically relevant symptomatology remains underexplored, warranting further investigation [21].

To fill these gaps, leveraging metabolomic data from the UK Biobank, we first identified and constructed a metabolic signature associated with UPF intake and assessed its associations with the incidence of overall mental disorder, depressive disorder, anxiety disorder, and substance use disorder (SUD). We then investigated the potential mediating role of the identified metabolic signature in the relationship between UPF exposure and mental health outcomes. We further examined the associations of UPF and its related metabolic alterations with specific psychological symptoms. We aimed to understand how UPF consumption contributes to the development of mental disorders and hypothesized that UPF-related metabolic alterations are associated with mental health outcomes and partially mediate the relationship between UPF intake and mental disorders. The results of this study therefore offered novel insights into the metabolic underpinnings of UPF-related neuropsychiatric risks, highlighting UPFs as a modifiable target for the primordial prevention of psychiatric disorders.

## 2. Materials and Methods

### 2.1. Study Population

This study utilized data from the UK Biobank, a large-scale prospective cohort comprising over 500,000 participants aged 37–73 years recruited across the United Kingdom between 2006 and 2010. The UK Biobank collected extensive data on sociodemographic, lifestyle, and biological factors, including untargeted metabolomic profiling and online dietary questionnaires. Participants were excluded if they lacked dietary data and metabolomic measurements, had a prior diagnosis of any mental disorder at baseline, or were lost to follow-up. After exclusions, a total of 30,059 participants were included in the final analysis, of whom 12,171 had completed the online mental health questionnaire (response rate: 42.3%). The UK Biobank study received ethical approval from the North West Multi-centre Research Ethics Committee, and all participants provided written informed consent. Data access for this study was granted under application number 99001. The present study adhered to the Strengthening the Reporting of Observational Studies in Epidemiology (STROBE) guidelines.

### 2.2. Exposure Assessment

Dietary intake information was obtained using the Oxford WebQ, an online self-administered 24 h dietary assessment tool specifically developed for large-scale population-based studies [22]. The tool captures detailed information on the types and quantities of approximately 200 commonly consumed foods and beverage items, enabling the estimation of total energy and nutrient intake. Within the past 24 h, individuals were instructed to report all food and drink they had ingested. The Oxford WebQ was implemented between 2011 and 2012, during which time most participants completed the questionnaire one to four times, with repeated assessments typically conducted every 3–4 months. Ultra-processed foods (UPFs) were classified using the NOVA system, which distinguishes food items into four categories depending on their level and intent of processing: (1) unprocessed or minimally processed foods, (2) processed culinary ingredients, (3) processed foods, and (4) UPFs [10]. In this study, UPFs were defined and quantified across eight distinct categories frequently observed in the UK Biobank dietary data: (1) beverages, (2) dairy products, (3) fruits and vegetables, (4) meat, fish, and eggs, (5) sauces and soups, (6) starchy foods, (7) sugary snacks, and (8) savory snacks (Supplementary Table S1). Total UPF intake was calculated as the summed weight of all UPF categories mentioned above (grams per day). To allow for comparability across individuals, UPF intake was then standardized as a proportion (%) of the total weight of all reported food and drink items (grams per day). As there are currently no established dietary guidelines for UPF intake, we further classified participants into low, moderate, and high UPF consumers based on tertiles of UPF intake.

### 2.3. Metabolic Profiling

Circulating metabolic profiles were measured using high-throughput nuclear magnetic resonance (NMR) spectroscopy performed by Nightingale Health Ltd. on plasma samples collected during the baseline assessment from a subset of 248,288 participants in the UK Biobank [23]. The platform quantified 251 metabolic biomarkers, encompassing 170 directly measured metabolites and 81 ratios of them. All samples were collected under standardized protocols and processed centrally to ensure consistency and quality control, as previously described [24]. Prior to all analyses, all metabolite values underwent logarithmic transformation followed by standardization into z-scores. Further details of the metabolites are in Supplementary Table S2.

### 2.4. Outcome

The primary outcomes of this study included mental disorder, depressive disorder, anxiety disorder, and SUD. These outcomes were ascertained through electronic health records within the UK Biobank. The diagnosis of each mental disorder was identified using the International Classification of Diseases Ninth and Tenth Revisions (ICD-9 and ICD-10) codes. Specific codes are provided in Supplementary Table S3. Follow-up time was calculated from the date of baseline assessment to the first diagnosis of the outcome of interest, death, or 19 December 2022, whichever came first.

The secondary outcomes were nineteen specific psychological symptoms assessed via the online mental health questionnaire, which was completed by a subset of participants (*n* = 12,171) between 2016 and 2017, including indicators of subjective well-being, depression, and anxiety. Subjective well-being was measured using three self-reported items addressing participants’ overall happiness, satisfaction with life, and feelings of meaning or purpose. Depressive and anxiety-related symptoms were assessed using the Patient Health Questionnaire-9 (PHQ-9) and Generalized Anxiety Disorder-7 (GAD-7) questionnaires. Each item was rated on a 4-point Likert scale ranging from 0 (“not at all”) to 3 (“nearly every day”) [25,26]. For each symptom, a response score of ≥1 was considered indicative of symptom presence. Detailed information on each symptom can be found in Supplementary Table S4.

### 2.5. Covariates

Potential confounders were selected based on the prior literature, including age, sex, index of multiple deprivation (IMD), body mass index (BMI), waist-to-hip ratio (WHR), healthy lifestyles (never smoking, moderate drinking, healthy sleep pattern, regular physical activity, and healthy diet) and prevalent diseases (hypertension, diabetes, and coronary artery disease) [21,27,28]. Briefly, IMD, reflecting socioeconomic status across geographic areas, was derived from composite scores based on seven domains and categorized as low (<12.16) or high (≥12.16). BMI was calculated from anthropometric measurements of weight and height. WHR was measured and calculated as waist circumference divided by hip circumference and classified as high (≥0.90 for men; ≥0.85 for women) or low (<0.90 for men; <0.85 for women) [29]. Participants were considered never smokers if they had never smoked, excluding both current and former smokers. Moderate alcohol consumption was defined as an average daily intake of no more than 16 g of pure alcohol. A healthy sleep pattern required meeting at least four of five favorable sleep characteristics, including early chronotype, 7–8 h of sleep per night, low frequency of insomnia and daytime sleepiness, and absence of snoring [30,31]. Regular physical activity was determined by adherence to the UK 2017 guidelines. Dietary habits were categorized as healthy if participants fulfilled at least four out of seven nutritional targets [32]. More information is available in Supplementary Table S5.

### 2.6. Statistical Analysis

Baseline characteristics are presented using means and standard deviations (SD) or medians with interquartile ranges (IQR) for continuous variables, and frequencies with percentages for categorical variables (Figure S1). To identify metabolic features associated with UPF intake, we employed an elastic net regression model using the standardized concentrations of 251 metabolites as predictors and UPF intake as the outcome variable. To identify the ideal value for the regularization parameter, a 10-fold cross-validation approach was applied. Metabolites with non-zero coefficients were retained, and a combined metabolic profile was then calculated by summing these metabolites, each multiplied by its corresponding regression-derived weight. The aggregate score was standardized and also categorized into tertiles, representing low, moderate, and high levels.

Cox proportional hazards regression models were applied to examine the associations of both UPF intake and the derived metabolic signature with incident mental disorders. Hazard ratios (HRs) and 95% confidence intervals (CIs) were estimated for both continuous and categorical variables, using follow-up time as the underlying time scale. The proportional hazards assumption was confirmed using Schoenfeld residual tests. Three different models were developed to estimate the associations. Model 1 (basic model) adjusted for age, sex, IMD, and BMI. Model 2 (multivariable model) included additional adjustments for WHR, lifestyle factors (never smoking, moderate drinking, healthy sleep pattern, regular physical activity, and healthy diet), and prevalent diseases (hypertension, diabetes, and coronary artery disease). Model 3 (mutually adjusted model) included both ultra-processed food intake and its corresponding metabolic signature to explore the independence of their associations with mental health outcomes. Moreover, logistic regression models were used to examine the associations of UPF intake and its metabolic signature with specific psychological symptoms, with odds ratios (ORs) and 95% CIs estimated. All models were adjusted for the same covariates as in the primary analyses. Stratified analyses were conducted by age (<60 years and ≥60 years) and sex (female and male) to examine whether the associations varied across demographic subgroups. Potential effect modification was evaluated through the inclusion of interaction terms in the models. Mediation analysis was conducted to evaluate whether the metabolic signature mediated the association between UPF intake and mental health outcomes. The mediation proportions were estimated using bootstrap methods with 1000 resamples for confidence interval estimation.

Multiple sensitivity analyses were conducted to evaluate the robustness of the results. First, absolute UPF intake (grams per day) was used in place of proportional intake. Second, analyses were repeated after excluding participants of non-White ethnicity to reduce potential confounding by race. Third, to avoid COVID-19-related bias, the follow-up period was truncated to 31 December 2019. Fourth, participants diagnosed with any of the outcomes within the first two years of follow-up were excluded to minimize reverse causation. Fifth, additional adjustments were made for environmental exposures including proximity to major roadways, traffic noise, ambient nitrogen dioxide (NO_2_), and fine particulate matter (PM_2.5_). Finally, Fine-Gray sub-distribution hazard models were applied to account for competing risks such as mortality that could preclude the occurrence of mental disorders [33]. All statistical analyses were performed using R 4.4.1, with significance determined by two-sided *p*-values less than 0.05, and false discovery rate (FDR) corrections were applied for multiple tests.

## 3. Results

**Figure 1:**
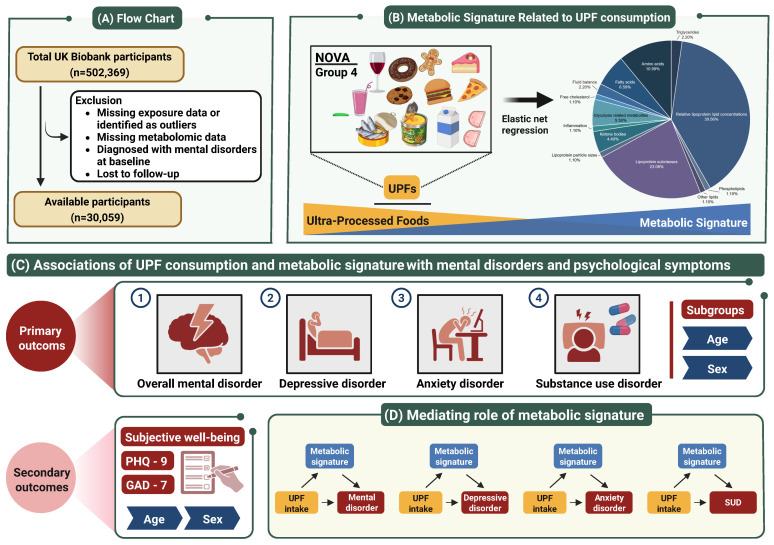
Study workflow. A total of 30,059 UK Biobank participants were included in this study, with a median follow-up of 12.6 years. (**A**) Study flowchart detailing participant selection, exclusion criteria, and final cohort. (**B**) Definition of ultra-processed food (UPF) intake and the composition of constructed metabolic signature. (**C**) Cox proportional hazards regression models were employed to evaluate associations of UPF intake and its metabolic signature with the incident risks of overall mental disorder, depressive disorder, anxiety disorder, and substance use disorder, with hazard ratios (HRs) and 95% confidence intervals (CIs) estimated. Logistic regression models were employed to evaluate associations of UPF intake and its metabolic signature with the risks of specific psychological symptoms, with odds ratios (ORs) and 95% CIs estimated. Subgroup analyses were conducted stratified by age and sex. (**D**) Mediation analyses were applied to investigate the mediating effect of UPF-related metabolic signature. SUD indicates substance use disorder.

After excluding individuals with missing dietary data and metabolomics measurements, prevalent psychiatric diseases, or loss to follow-up, a total of 30,059 participants were included in the final analysis (Figure 1). The mean age of participants was 56.5 years, and 53.1% were female. Over a median follow-up of 12.6 years, 7594 participants developed mental disorders (7594 participants of mental disorder, 865 participants of depressive disorder, 892 participants of anxiety disorder, and 1300 participants of SUD) with the incidence rates of 2275.2, 229.9, 236.9, and 348.9 (per 100,000 person-years), respectively (Supplementary Table S6). In the study population, the mean (SD) proportion of UPF intake was 15.6% (11.1%). Baseline characteristics of participants, classified by UPF consumption level, are presented in Table 1. Notably, participants with higher UPF consumption levels tended to be younger, have higher BMI, high WHR, higher socioeconomic status, and unhealthy lifestyles.

**Figure 2:**
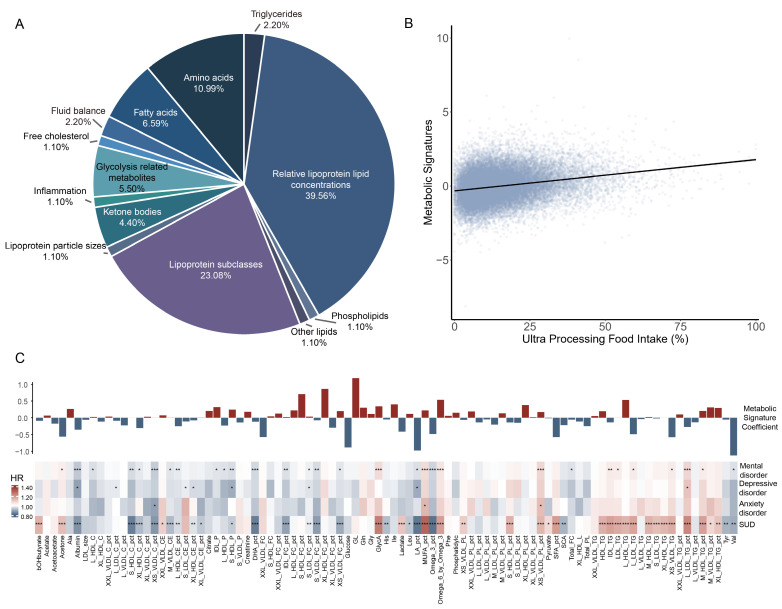
Constructions of the metabolic signature. (**A**) The composition of the metabolic signature for ultra-processed food intake. (**B**) The correlations between ultra-processed food intake and the metabolic signature. (**C**) From top to bottom are the metabolites’ coefficients (weights) and associations between 91 metabolites constituting the metabolic signature and mental disorders. Colors depict the direction of associations (positive-red and inverse-blue); asterisks denote significance levels (* *p* FDR < 0.05, ** *p* FDR < 0.01, and *** *p* FDR < 0.001). Ala indicates Alanine; bOHbutyrate, 3-Hydroxybutyrate; C, cholesterol; CE, cholesteryl ester; DHA, docosahexaenoic acid; FC, free cholesterol; GL, glucose-lactate; Gln, glutamine; Gly, glycine; GlycA, glycoprotein acetyls; His, histidine; HR, hazard ratio; Ile, isoleucine; L, large; LA, linoleic acid; Leu, leucine; LDL, low-density lipoprotein; M, medium; MUFA, monounsaturated fatty acids; P, particles; Phe, phenylalanine; PL, phospholipid; S, small; SFA, saturated fatty acids; SCA, spectrometer-corrected alanine; SUD, substance use disorder; TG, triglycerides; Tyr, tyrosine; Val, valine; VLDL, very LDL; XL, very large; XS, very small; XXL, extra large.

Using elastic net regression, we identified 91 metabolites associated with UPF intake. These metabolites spanned diverse biochemical categories encompassing fatty acids, lipoproteins, and other small molecules. Among them, the free cholesterol to total lipids ratio in very large HDL, glucose-lactate composite, the ratio of linoleic acid to total fatty acids, and valine exhibited the largest contribution to the metabolic signature, indicating their potential relevance in UPF-related metabolic alterations. Based on these findings, a metabolite profile score was constructed to capture the metabolic signature of UPF intake, providing a composite biomarker reflective of its systemic metabolic impact (Figure 2).

**Figure 3:**
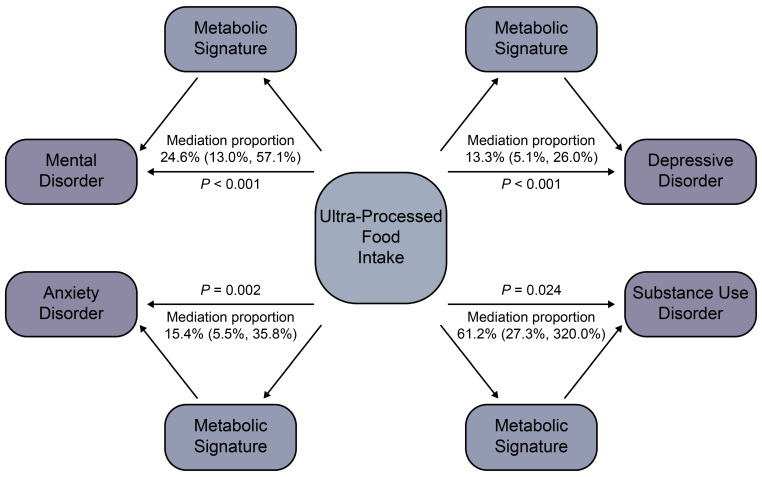
The mediating effects of the metabolic signature on the association between ultra-processed food intake and mental disorder risk. Mediation analysis was conducted to evaluate whether the metabolic signature mediated the association between ultra-processed food intake and mental health outcomes. The mediation proportions were estimated using bootstrap methods with 1000 resamples for confidence interval estimation.

Cox proportional hazard models were used to assess the associations of UPF intake and its corresponding metabolic signature with mental disorders (Table 2). In the multivariable models, compared with participants with low UPF intake, participants with high UPF intake exhibited increased risks of mental disorder, depressive disorder, and anxiety disorder, with HRs (95% CIs) of 1.08 (1.02, 1.14), 1.35 (1.14, 1.60), and 1.32 (1.12, 1.56), respectively. As for continuous variables, higher UPF intake was positively associated with mental disorder, depressive disorder, anxiety disorder, and SUD, with HRs (95% CIs) per 10% increment of 1.04 (1.02, 1.06), 1.14 (1.08, 1.20), 1.12 (1.06, 1.18), and 1.06 (1.01, 1.11), respectively. Similarly, a higher UPF-related metabolic signature was associated with increased risks of all four mental health outcomes when comparing participants with high scores versus those with low scores, with HRs (95% CIs) of 1.18 (1.11, 1.25), 1.32 (1.10, 1.58), 1.37 (1.15, 1.63), and 1.60 (1.39, 1.85), respectively. When modeled as a continuous variable, the metabolic signature was associated with elevated risks of mental disorder, depressive disorder, anxiety disorder, and SUD, with HRs per SD increment (95% CIs) of 1.08 (1.05, 1.10), 1.16 (1.08, 1.24), 1.15 (1.07, 1.23), 1.25 (1.19, 1.32), respectively. The robustness of the associations was confirmed in the mutually adjusted models. Furthermore, mediation analyses indicated that the UPF-related metabolic signature partially mediated the associations between UPF intake and the risks of mental disorders with mediation proportions (95% CIs) of 24.6% (13.0%, 57.1%) for mental disorder, 13.3% (5.1%, 26.0%) for depressive disorder, 15.4% (5.5%, 35.8%) for anxiety disorder, and 61.2% (27.3%, 320.0%) for SUD (Figure 3). Subgroup analyses indicated that adults under 60 were particularly susceptible to elevated consumption of UPFs for SUD (*p* for interaction = 0.009) (Supplementary Tables S7 and S8). A similar pattern was observed for the UPF-related metabolic signature, with stronger associations among individuals < 60 years for depressive disorder (*p* for interaction = 0.036) and anxiety disorder (*p* for interaction = 0.021). Furthermore, the link between the UPF-related metabolic signature and SUD was more evident in females than males (*p* for interaction = 0.004) (Supplementary Tables S9 and S10).

**Figure 4:**
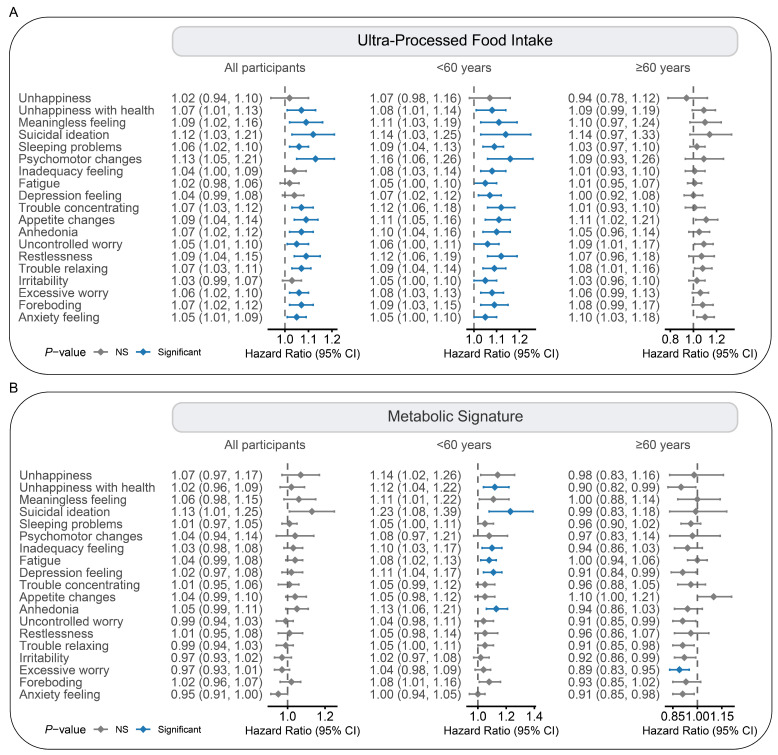
Associations of ultra-processed food intake and its metabolic signature with symptoms of mental health and stratified by age. The symptoms included three categories, namely subjective well-being (3 items), PHQ-9 (9 items), and GAD-7 (7 items). Logistic regression models were employed to evaluate associations of ultra-processed food (UPF) intake and its metabolic signature with the risks of specific psychological symptoms. (**A**) For UPF intake. (**B**) For metabolic signature. The results were presented with odds ratios (ORs) per 10% increment for UPF intake and ORs per SD increment for metabolic signature. Colors depict significance levels after false discovery rate correction (significant-blue and not significant-gray).

Logistic regression models were further used to examine the associations of UPF intake and its corresponding metabolic signature with mental health symptoms. In the multivariable models, higher UPF intake was associated with increased risks of several mental health symptoms, including unhappiness with health, suicidal ideation, and anxiety feeling, with ORs (95% CIs) per 10% increment of 1.07 (1.01, 1.13), 1.12 (1.03, 1.16), and 1.05 (1.01, 1.09), respectively. In contrast, the UPF-related metabolic signature showed no significant associations with mental health symptoms in the overall population (Figure 4). However, subgroup analyses revealed that individuals under 60 years were affected by the metabolic signature for several mental health symptoms, including unhappiness with health (*p* for interaction = 0.002) and depressive feeling (*p* for interaction = 0.002). No significant effect modification was observed by sex (Supplementary Tables S11–S14). Sensitivity analyses confirmed the robustness of the associations above (Supplementary Table S15).

## 4. Discussion

In this large-scale cohort study involving over 30,000 participants, we found that UPF intake was significantly associated with increased risks of mental disorder, depressive disorder, anxiety disorder, and SUD. By leveraging NMR metabolomic profiling, we identified a metabolic signature comprising 91 metabolites linked to UPF intake and constructed a composite score reflecting its systemic metabolic impact. This UPF-related metabolic signature was also independently associated with elevated risks of these mental disorders, and notably, partially mediated the relationship between UPF intake and psychiatric diseases. Furthermore, significant effect modifications by age and sex were observed, with stronger associations in individuals under 60 years and in females for certain outcomes. Our findings were further supported by consistent results in analyses of self-reported mental health symptoms. Collectively, these results confirmed the proposed hypotheses, demonstrating that UPF-related metabolic alterations are linked to mental disorders and mediate, at least in part, the association between UPF intake and psychiatric outcomes.

Previous epidemiological studies have brought growing attention to the possible impacts of UPF consumption on mental health [15,16,34]. For example, a prospective cohort study followed over 15,000 adults in Brazil found that higher UPF intake predicted an increased risk of mental disorder (Relative Risk [RR] per SD increase: 1.06; 95% CI: 1.01–1.12), and anxiety disorder (RR: 1.09; 95% CI: 1.02–1.16) [15]. In another prospective study conducted within the Nurses’ Health Study II among middle-aged females, greater intake of UPFs was linked to a heightened risk of depressive disorder, with those in the top quintile showing a 34% higher risk compared to those in the lowest quintile (HR: 1.34; 95% CI: 1.20–1.50) [16]. Evidence from a cohort study in the UK Biobank also suggested that high UPF consumption was significantly associated with an increased risk of depressive disorder (HR: 1.83; 95% CI: 1.45–2.31) [34]. Despite the growing body of evidence, previous investigations have largely emphasized the links between UPFs and diagnosed mental health outcomes, and few have explored potential biological mechanisms underlying these associations.

One promising area of investigation involves metabolic dysregulation, which has gained growing recognition as a major factor influencing the onset and advancement of psychiatric disorders [35,36,37,38]. For instance, a large-scale metabolomics study conducted in a Dutch clinical cohort identified a comprehensive metabolic profile of major depressive disorder (MDD), characterized by reduced levels of long-chain fatty acids alongside increased concentrations of lysophospholipids, which was further supported by Mendelian randomization analyses [39]. Complementary evidence from a cross-sectional study has further demonstrated altered plasma metabolism in MDD adolescents compared to healthy controls, with distinctive changes observed in amino acid pathways such as lysine, cysteine, and methionine metabolism [40]. These findings suggested that metabolic health is intricately connected to brain function and emotional regulation, providing a plausible mechanistic link through which dietary exposures, including UPF intake, may impact mental well-being. In this context, our findings extend the current literature by confirming the association between UPF intake and a broader spectrum of psychiatric disorders and mental health symptoms, and by providing novel insights into the metabolic perturbations that may mediate these effects.

Researchers have put forward several mechanistic explanations for the relationship between UPF intake and mental disorders. UPFs are typically characterized by high levels of added sugars, unhealthy fats, sodium, synthetic additives, and low content of essential nutrients and dietary fiber. Moreover, the intense processing of UPFs often leads to the creation of novel food matrices and bio-incompatible compounds, such as advanced glycation end products (AGEs) and neo-formed contaminants, which may exert pro-inflammatory and neurotoxic effects upon chronic exposure [41]. Such compositions might promote oxidative damage and neuroinflammation, both of which are linked to the pathogenesis of mental disorders [42]. Simultaneously, excessive consumption of UPFs can disturb the balance of gut microbial communities and compromise intestinal barrier integrity, thereby inducing systemic inflammation and altering neurochemical signaling pathways [43,44,45]. Furthermore, chronic exposure to UPFs has shown ties to impaired dysregulation of the HPA axis and neuroendocrine imbalances, including insulin resistance and leptin dysfunction, which might interfere with stress regulation and emotional processing [42,46]. Our identification of the metabolic signature associated with UPF intake provides further support for these mechanisms. Notably, key metabolites in this signature, such as altered lipoprotein profiles, elevated glucose-lactate composite, and changes in amino acid metabolism (e.g., valine), may reflect systemic metabolic disturbances that influence neurobiological pathways related to mood regulation, stress response, and addiction vulnerability. These findings underscore the role of nutrition-related metabolic alterations as potential mediators linking UPF consumption to adverse mental health outcomes.

The observed effect modifications by age and sex offer important insights into population vulnerability to UPF-related mental health risks. Our findings suggest that middle-aged individuals (<60 years) may be more susceptible to both UPF intake and its associated metabolic disturbances. This heightened vulnerability might reflect greater exposure to lifestyle stressors, higher baseline metabolic burden, or greater sensitivity to diet-induced neuroinflammation during midlife [1]. Furthermore, the stronger associations between the UPF-related metabolic signature and SUD in females highlight potential sex-based differences in metabolic regulation, hormonal influences, and neurobiological response to addictive behaviors [47]. These findings underscore the importance of considering demographic context when evaluating dietary risk factors for mental disorders. In light of this, personalized prevention strategies may be warranted to mitigate the mental health burden associated with high UPF consumption.

Over the past few decades, the consumption of UPFs has increased dramatically across the globe, reshaping dietary patterns in both developed and developing countries. During this ongoing global transition, traditional diets rich in whole and minimally processed foods are being rapidly displaced by highly industrialized food products [48,49,50]. This shift has led to increasingly homogenized food environments, where convenience, shelf life, and branding often override nutritional quality [12,51]. From a public health perspective, this global surge in UPF consumption raises substantial concerns [52,53]. However, although UPFs have been well-documented as contributing to obesity, diabetes, and cardiovascular diseases, public awareness of their potential impact on mental health and overall well-being remains limited. In this context, urgent action is needed to counteract this global dietary shift, reshape public perceptions, and mitigate its impact on public health, including mental health. Effective approaches might include revising national dietary guidelines to explicitly limit UPF consumption, implementing stricter marketing restrictions, and expanding subsidies or incentives to increase the availability and affordability of fresh, minimally processed foods [54,55]. Moreover, doctors and nutritionists working in hospitals and primary care settings could play a pivotal role in promoting healthy eating behaviors, by providing dietary counseling, screening for high UPF consumption, and offering tailored nutritional interventions [56]. Nutrition education programs led by communities and schools could further support these efforts by helping individuals develop the knowledge and skills needed to identify UPFs and understand their health impacts.

Several limitations of this study should be acknowledged. First, the cohort was predominantly composed of individuals of White European ancestry from the UK, which may limit the generalizability of our findings to other ethnic groups or populations with different cultural and dietary backgrounds. Future studies in more ethnically diverse cohorts and with repeated dietary and metabolic measurements are warranted to validate and extend our findings. Second, mental disorder outcomes were identified through linked health records, which may underestimate milder or undiagnosed cases, particularly for conditions like anxiety and depression. To address this limitation, we additionally examined individual mental health symptoms to capture subtle changes in psychological states that may not meet diagnostic standards [57]. Third, although we adjusted for a wide range of sociodemographic and lifestyle factors, residual confounding cannot be fully excluded [58,59]. We also conducted a sensitivity analysis with additional adjustments for environmental factors to further minimize potential sources of bias. Fourth, while the metabolomic profiling enabled us to explore potential biological mechanisms, the cross-sectional nature of the metabolite data limits causal inference regarding temporal relationships. Fifth, dietary intake was assessed at baseline using self-reported food frequency questionnaires, which may be subject to recall bias and misclassification [60]. Self-reported mental health data might also introduce similar biases. Future studies incorporating precise dietary and psychological assessments would help validate our results. Sixth, the UK Biobank cohort comprises individuals who are generally healthier than the general population, and within the cohort, those who completed the mental health questionnaire tend to be even healthier, potentially introducing voluntary response bias (Table S16). Lastly, although the NOVA classification system was applied to categorize UPFs, some degree of misclassification is possible due to limited granularity in food item descriptions.

## 5. Conclusions

In conclusion, higher consumption of UPFs was associated with an increased risk of mental health problems, including anxiety, depressive, and substance use disorders. We also found that changes in blood metabolite patterns linked to UPF intake might help explain these associations. These findings suggest that reducing UPF intake and improving diet quality might help maintain mental well-being. Moreover, our findings illustrated the potential of metabolomics as a powerful tool in precision medicine, enabling the identification of at-risk individuals and the development of tailored interventions to promote mental well-being. Future research should explore the metabolic signature in different populations and validate the causal pathways through clinical trials and animal experiments.

## Figures and Tables

**Table 1:** Baseline characteristics of participants grouped by ultra-processed food intake level.

Characteristics	All Participants	Ultra-Processed Food Intake Level ^a^			*p* Value ^b^
		Low (*n* = 19,455)	Moderate (*n* = 19,454)	High (*n* = 19,514)	
Age	56.5 (8.1)	57.1 (7.8)	57.1 (7.8)	55.3 (8.4)	<0.001
Sex					<0.001
Female	15,956 (53.1%)	5446 (54.4%)	5359 (53.5%)	5151 (51.3%)	
Male	14,103 (46.9%)	4564 (45.6%)	4650 (46.5%)	4889 (48.7%)	
IMD ^c^					<0.001
Low	15,041 (50.0%)	5221 (52.2%)	5194 (51.9%)	4626 (46.1%)	
High	15,018 (50.0%)	4789 (47.8%)	4815 (48.1%)	5414 (53.9%)	
BMI	27.1 (4.7)	26.6 (4.4)	26.8 (4.4)	27.9 (5.0)	<0.001
WHR ^d^					<0.001
Low	15,578 (51.8%)	5471 (54.7%)	5312 (53.1%)	4795 (47.8%)	
High	14,481 (48.2%)	4539 (45.3%)	4697 (46.9%)	5245 (52.2%)	
Healthy lifestyle					
Never smoking ^e^	17,485 (58.2%)	5564 (55.6%)	5959 (59.5%)	5962 (59.4%)	<0.001
Moderate drinking ^f^	20,277 (67.5%)	6136 (61.3%)	6782 (67.8%)	7359 (73.3%)	<0.001
Regular physical activity ^g^	16,717 (55.6%)	5735 (57.3%)	5592 (55.9%)	5390 (53.7%)	<0.001
Healthy sleep pattern ^h^	16,930 (56.3%)	5792 (57.9%)	5669 (56.6%)	5469 (54.5%)	<0.001
Healthy diet ^i^	11,708 (39.0%)	4603 (46.0%)	3913 (39.1%)	3192 (31.8%)	<0.001
Prevalent disease					
Hypertension	7943 (26.4%)	2613 (26.1%)	2566 (25.6%)	2764 (27.5%)	0.007
Diabetes	1378 (4.6%)	443 (4.4%)	383 (3.8%)	552 (5.5%)	<0.001
Coronary artery disease	606 (2.0%)	197 (2.0%)	191 (1.9%)	218 (2.2%)	0.381

Abbreviations: IMD, index of multiple deprivation; BMI, body mass index; WHR, waist-to-hip ratio. ^a^ Participants’ ultra-processed food intakes were categorized into tertiles based on the distribution, classified as low, moderate, and high. ^b^ Group comparisons were conducted using analysis of variance (ANOVA) or the chi-square (χ^2^) test, as appropriate. ^c^ IMD was calculated through indicators across several different domains, including crime score, community safety score, education score, employment score, health score, housing score, income score, living environment score, access to services score, and physical environment score, and was categorized as low (<12.16) and high (≥12.16). ^d^ The WHR was calculated as waist circumference (cm) divided by hip circumference (cm) and classified as low (<0.9 for men and <0.85 for women) or high (≥0.9 for men and ≥0.85 for women). ^e^ Never smoking was defined as the absence of both past and current smoking at baseline. ^f^ Moderate drinking was defined as an average daily intake of ≤16 g of pure alcohol (equivalent to ≤2 units) for both men and women. ^g^ Regular physical activity was defined as engagement in at least 150 min of moderate-intensity activity per week, at least 75 min of vigorous-intensity activity per week, or an equivalent combination of both. ^h^ A healthy sleep pattern was assessed based on five dimensions of sleep behavior: early chronotype, sleep duration of 7–8 h per day, absence of frequent insomnia (never/rarely or sometimes), no self-reported snoring, and absence of excessive daytime sleepiness (never/rarely or sometimes). Participants meeting at least four of these five criteria were classified as having a healthy sleep pattern. ^i^ A healthy diet was defined as the consumption of at least four out of seven key food groups prioritized for cardiometabolic health. The specific criteria for each component were as follows: ≥3 servings/day of fruits, ≥3 servings/day of vegetables, ≥2 servings/week of fish, ≤1 serving/week of processed meats, ≤1.5 servings/week of unprocessed red meats, ≥3 servings/day of whole grains, and ≤1.5 servings/day of refined grains.

**Table 2:** Associations of ultra-processed food intake and mental disorders.

Disease	Categorical ^a^				Continuous ^b^	
	Low	Moderate	High	*p* Trend	HR (95% CI)	*p* Value
Ultra-processed food intake						
Mental disorder						
Basic ^c^	reference	1.02 (0.96, 1.08)	1.08 (1.02, 1.14)	0.006	1.04 (1.02, 1.07)	<0.001
MV ^d^	reference	1.03 (0.97, 1.09)	1.08 (1.02, 1.14)	0.011	1.04 (1.02, 1.06)	<0.001
MV + MA ^e^	reference	1.02 (0.96, 1.07)	1.05 (1.00, 1.12)	0.068	1.03 (1.01, 1.05)	0.006
Depressive disorder						
Basic	reference	1.12 (0.94, 1.33)	1.39 (1.18, 1.65)	<0.001	1.16 (1.10, 1.22)	<0.001
MV	reference	1.12 (0.94, 1.33)	1.35 (1.14, 1.60)	<0.001	1.14 (1.08, 1.20)	<0.001
MV + MA	reference	1.10 (0.92, 1.31)	1.31 (1.10, 1.55)	0.002	1.12 (1.06, 1.18)	<0.001
Anxiety disorder						
Basic	reference	1.17 (0.99, 1.38)	1.35 (1.15, 1.59)	<0.001	1.13 (1.07, 1.19)	<0.001
MV	reference	1.16 (0.98, 1.38)	1.32 (1.12, 1.56)	0.001	1.12 (1.06, 1.18)	<0.001
MV + MA	reference	1.14 (0.96, 1.35)	1.28 (1.08, 1.51)	0.005	1.10 (1.04, 1.16)	0.001
SUD						
Basic	reference	0.95 (0.83, 1.09)	0.96 (0.84, 1.10)	0.53	1.04 (0.99, 1.09)	0.100
MV	reference	1.03 (0.90, 1.18)	1.03 (0.90, 1.18)	0.684	1.06 (1.01, 1.11)	0.020
MV + MA	reference	1.00 (0.87, 1.15)	0.96 (0.84, 1.11)	0.597	1.02 (0.97, 1.07)	0.377
Metabolic signature score						
Mental disorder						
Basic	reference	1.08 (1.02, 1.14)	1.23 (1.16, 1.30)	<0.001	1.10 (1.07, 1.12)	<0.001
MV	reference	1.07 (1.01, 1.13)	1.18 (1.11, 1.25)	<0.001	1.08 (1.05, 1.10)	<0.001
MV + MA	reference	1.07 (1.01, 1.13)	1.17 (1.10, 1.24)	<0.001	1.07 (1.04, 1.10)	<0.001
Depressive disorder						
Basic	reference	1.13 (0.94, 1.34)	1.44 (1.21, 1.71)	<0.001	1.21 (1.13, 1.30)	<0.001
MV	reference	1.10 (0.92, 1.31)	1.32 (1.10, 1.58)	0.002	1.16 (1.08, 1.24)	<0.001
MV + MA	reference	1.07 (0.90, 1.28)	1.27 (1.06, 1.52)	0.008	1.13 (1.05, 1.21)	0.001
Anxiety disorder						
Basic	reference	1.19 (1.00, 1.41)	1.44 (1.21, 1.71)	<0.001	1.18 (1.10, 1.26)	<0.001
MV	reference	1.17 (0.98, 1.38)	1.37 (1.15, 1.63)	<0.001	1.15 (1.07, 1.23)	<0.001
MV + MA	reference	1.14 (0.96, 1.36)	1.32 (1.10, 1.57)	0.002	1.12 (1.05, 1.21)	0.001
SUD						
Basic	reference	1.02 (0.88, 1.19)	1.61 (1.40, 1.86)	<0.001	1.28 (1.21, 1.35)	<0.001
MV	reference	1.05 (0.90, 1.23)	1.60 (1.39, 1.85)	<0.001	1.25 (1.19, 1.32)	<0.001
MV + MA	reference	1.06 (0.91, 1.23)	1.61 (1.39, 1.86)	<0.001	1.25 (1.18, 1.32)	<0.001

Abbreviations: SUD, Substance use disorder; HR, hazard ratio; CI, confidence interval. ^a^ Participants’ ultra-processed food intake and related metabolic signature were categorized into tertiles based on the distribution and classified as low, moderate, and high levels, with the low level serving as the reference group. ^b^ Ultra-processed food intake was analyzed as a continuous variable with per 10% increment, and metabolic signature was analyzed as a continuous variable with per SD increment. ^c^ Basic model adjusted for age, sex, IMD, and BMI. ^d^ Multivariable model (MV) additionally adjusted for WHR, healthy lifestyle factors, and prevalent diseases. ^e^ Mutually adjusted model (MV + MA) included both ultra-processed food intake and its corresponding metabolic signature.

## Data Availability

All the data for this study will be made available upon reasonable request to the corresponding authors.

## Supplementary Materials

The following supporting information can be downloaded at: https://www.mdpi.com/article/10.3390/nu17091582/s1, Table S1: Examples of ultra-processed foods according to the NOVA classification; Table S2: Details of metabolites in UK Biobank; Table S3: Definitions of primary outcomes; Table S4: Definitions of secondary outcomes; Table S5: Definitions of baseline characteristics and healthy lifestyles; Table S6: New events, total person-years and incidence rates of mental disorders; Table S7: Associations of ultra-processed food intake level and mental disorders by age and sex; Table S8: Associations of ultra-processed food intake and mental disorders by age and sex; Table S9: Associations of metabolic signature score level and mental disorders by age and sex; Table S10: Associations of metabolic signature score and mental disorders by age and sex; Table S11: Associations of ultra-processed food intake and mental health symptoms by age; Table S12: Associations of ultra-processed food intake and mental health symptoms by sex; Table S13: Associations of metabolic signature score and mental health symptoms by age; Table S14: Associations of metabolic signature score and mental health symptoms by sex; Table S15: Sensitivity analysis of the main associations; Table S16. Baseline characteristics of participants grouped by completion of the mental health questionnaire.
