# Supplementary material for: Associations of Ultra-Processed Food Intake and Its Circulating Metabolomic Signature with Mental Disorders in Middle-Aged and Older Adults
Source: Nutrients. 2025 May 4;17(9):1582. doi: 10.3390/nu17091582 (PMC12073222; doi:10.3390/nu17091582)
Supplement: Supplementary file 1 [file nutrients-17-01582-s001.zip › nutrients-3622470-supplementary.pdf]

Supplementary Information

Associations of Ultra-Processed Food Intake and Its Circulating Metabolomic Signature with Mental Disorders in Middle-Aged and Older Adults

Figure S1. Normality test for the continuous variables..2

Table S1. Examples of ultra-processed foods according to the NOVA classification............................3

Table S2. Details of metabolites in UK Biobank..4

Table S3. Definitions of primary outcomes 11

Table S4. Definitions of secondary outcomes..12

Table S5. Definitions of baseline characteristics and healthy lifestyles 13

Table S6. New events, total person-years and incidence rates of mental disorders.............................15

Table S7. Associations of ultra-processed food intake level and mental disorders by age and sex......16

Table S8. Associations of ultra-processed food intake and mental disorders by age and sex..............17

Table S9. Associations of metabolic signature score level and mental disorders by age and sex.........18

Table S10. Associations of metabolic signature score and mental disorders by age and sex...............19

Table S11. Associations of ultra-processed food intake and mental health symptoms by age.............20

Table S12. Associations of ultra-processed food intake and mental health symptoms by sex.............21

Table S13. Associations of metabolic signature score and mental health symptoms by age................22

Table S14. Associations of metabolic signature score and mental health symptoms by sex 23

Table S15. Sensitivity analysis of the main associations 24

Table S16. Baseline characteristics of participants grouped by completion of the mental health questionnaire..26

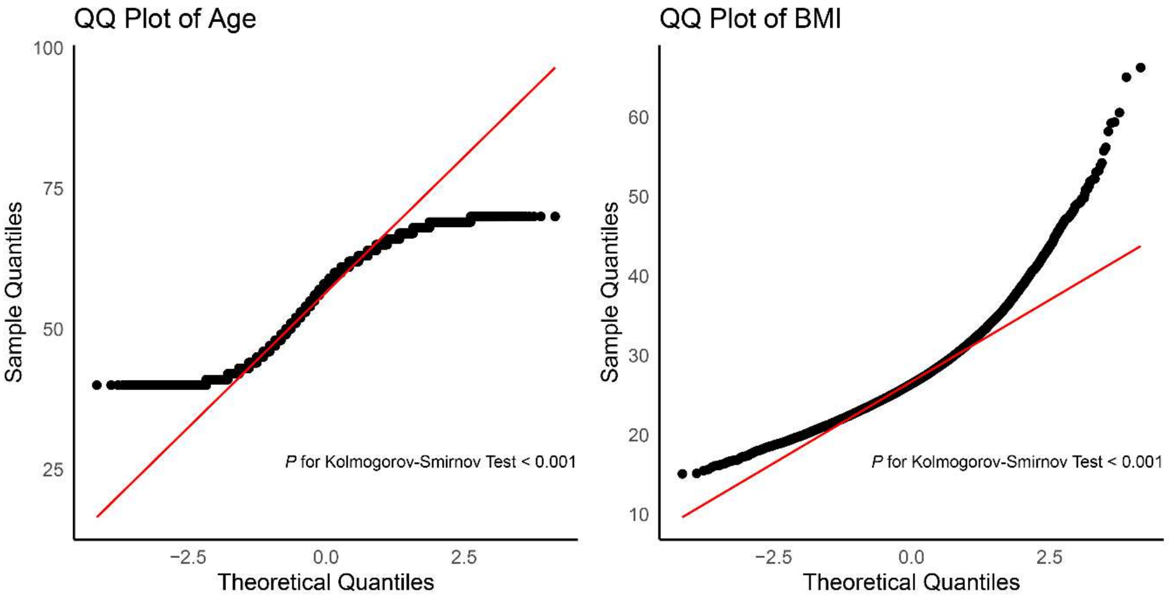

Figure S1. Normality test for the continuous variables

Table S1. Examples of ultra-processed foods according to the NOVA classification

Group	Examples of foods	UKB Data-Field
Beverages	Artificially sweetened beverages; distilled alcoholic beverages.	26126, 26127, 26138
Dairy products	Flavored milk, yogurt.	26086, 26087, 26102, 26084, 26124
Fruits and vegetables	Baked bean, tinned tomato.	26064, 26090, 26144
Meat, fish and egg	Bacon, ham, crumbed or deep-fried poultry, sausage; tinned tuna; eggs in sandwiches.	26122, 26137, 26145, 26149
Sauces and soups	Hummus, guacamole, tomato ketchup; powdered/instant soup, canned soup.	26106, 26129, 26130, 26110
Starchy foods	Oat crunch, flavored cereal, pizza, bread.	26075, 26068, 26076, 26119, 26105, 26078, 26116, 26073, 26079, 26097
Sugary snacks	Cakes, chocolate bars, ice cream, pudding.	26080, 26085, 26140
Savory snacks	Indian snacks, salted nuts, savory biscuits.	26108, 26083, 26134

Table S2. Details of metabolites in UK Biobank

Metabolites	Abbr	Group	Code
Total cholesterol	Total_C	Cholesterol	23400
Total cholesterol minus HDL-C	non_HDL_C	Cholesterol	23401
Remnant cholesterol (non-HDL, non-LDL -cholesterol)	Remnant_C	Cholesterol	23402
VLDL cholesterol	VLDL_C	Cholesterol	23403
Clinical LDL cholesterol	Clinical_LDL_C	Cholesterol	23404
LDL cholesterol	LDL_C	Cholesterol	23405
HDL cholesterol	HDL_C	Cholesterol	23406
Total triglycerides	Total_TG	Triglycerides	23407
Triglycerides in VLDL	VLDL_TG	Triglycerides	23408
Triglycerides in LDL	LDL_TG	Triglycerides	23409
Triglycerides in HDL	HDL_TG	Triglycerides	23410
Total phospholipids in lipoprotein particles	Total_PL	Phospholipids	23411
Phospholipids in VLDL	VLDL_PL	Phospholipids	23412
Phospholipids in LDL	LDL_PL	Phospholipids	23413
Phospholipids in HDL	HDL_PL	Phospholipids	23414
Total esterified cholesterol	Total_CE	Cholesteryl esters	23415
Cholesteryl esters in VLDL	VLDL_CE	Cholesteryl esters	23416
Cholesteryl esters in LDL	LDL_CE	Cholesteryl esters	23417
Cholesteryl esters in HDL	HDL_CE	Cholesteryl esters	23418
Total free cholesterol	Total_FC	Free cholesterol	23419
Free cholesterol in VLDL	VLDL_FC	Free cholesterol	23420
Free cholesterol in LDL	LDL_FC	Free cholesterol	23421
Free cholesterol in HDL	HDL_FC	Free cholesterol	23422
Total lipids in lipoprotein particles	Total_L	Total lipids	23423
Total lipids in VLDL	VLDL_L	Total lipids	23424
Total lipids in LDL	LDL_L	Total lipids	23425
Total lipids in HDL	HDL_L	Total lipids	23426
Total concentration of lipoprotein particles	Total_P	Lipoprotein particle concentrations	23427
Concentration of VLDL particles	VLDL_P	Lipoprotein particle concentrations	23428
Concentration of LDL particles	LDL_P	Lipoprotein particle concentrations	23429
Concentration of HDL particles	HDL_P	Lipoprotein particle concentrations	23430
Average diameter for VLDL particles	VLDL_size	Lipoprotein particle sizes	23431
Average diameter for LDL particles	LDL_size	Lipoprotein particle sizes	23432
Average diameter for HDL particles	HDL_size	Lipoprotein particle sizes	23433
Phosphoglycerides	Phosphoglyc	Other lipids	23434
Ratio of triglycerides to phosphoglycerides	TG_by_PG	Other lipids	23435
Total cholines	Cholines	Other lipids	23436
Phosphatidylcholines	Phosphatidylc	Other lipids	23437
Sphingomyelins	Sphingomyelins	Other lipids	23438

Apolipoprotein B	ApoB	Apolipoproteins	23439
Apolipoprotein A1	ApoA1	Apolipoproteins	23440
Ratio of apolipoprotein B to apolipoprotein A1	ApoB_by_ApoA1	Apolipoproteins	23441
Total fatty acids	Total_FA	Fatty acids	23442
Degree of unsaturation	Unsaturation	Fatty acids	23443
Omega-3 fatty acids	Omega_3	Fatty acids	23444
Omega-6 fatty acids	Omega_6	Fatty acids	23445
Polyunsaturated fatty acids	PUFA	Fatty acids	23446
Monounsaturated fatty acids	MUFA	Fatty acids	23447
Saturated fatty acids	SFA	Fatty acids	23448
Linoleic acid	LA	Fatty acids	23449
Docosahexaenoic acid	DHA	Fatty acids	23450
Ratio of omega-3 fatty acids to total fatty acids	Omega_3_pct	Fatty acids	23451
Ratio of omega-6 fatty acids to total fatty acids	Omega_6_pct	Fatty acids	23452
Ratio of polyunsaturated fatty acids to total fatty acids	PUFA_pct	Fatty acids	23453
Ratio of monounsaturated fatty acids to total fatty acids	MUFA_pct	Fatty acids	23454
Ratio of saturated fatty acids to total fatty acids	SFA_pct	Fatty acids	23455
Ratio of linoleic acid to total fatty acids	LA_pct	Fatty acids	23456
Ratio of docosahexaenoic acid to total fatty acids	DHA_pct	Fatty acids	23457
Ratio of polyunsaturated fatty acids to monounsaturated fatty acids	PUFA_by_MUFA	Fatty acids	23458
Ratio of omega-6 fatty acids to omega-3 fatty acids	Omega_6_by_Omega_3	Fatty acids	23459
Alanine	Ala	Amino acids	23460
Glutamine	Gln	Amino acids	23461
Glycine	Gly	Amino acids	23462
Histidine	His	Amino acids	23463
Total concentration of branched-chain amino acids (leucine + isoleucine + valine)	Total_BCAA	Amino acids	23464
Isoleucine	Ile	Amino acids	23465
Leucine	Leu	Amino acids	23466
Valine	Val	Amino acids	23467
Phenylalanine	Phe	Amino acids	23468
Tyrosine	Tyr	Amino acids	23469
Glucose	Glucose	Glycolysis related metabolites	23470
Lactate	Lactate	Glycolysis related metabolites	23471
Pyruvate	Pyruvate	Glycolysis related metabolites	23472
Citrate	Citrate	Glycolysis related metabolites	23473
3-Hydroxybutyrate	bOHbutyrate	Ketone bodies	23474
Acetate	Acetate	Ketone bodies	23475
Acetoacetate	Acetoacetate	Ketone bodies	23476
Acetone	Acetone	Ketone bodies	23477
Creatinine	Creatinine	Fluid balance	23478

Albumin	Albumin	Fluid balance	23479
Glycoprotein acetyls	GlycA	Inflammation	23480
Concentration of chylomicrons and extremely large VLDL particles	XXL_VLDL_P	Lipoprotein subclasses	23481
Total lipids in chylomicrons and extremely large VLDL	XXL_VLDL_L	Lipoprotein subclasses	23482
Phospholipids in chylomicrons and extremely large VLDL	XXL_VLDL_PL	Lipoprotein subclasses	23483
Cholesterol in chylomicrons and extremely large VLDL	XXL_VLDL_C	Lipoprotein subclasses	23484
Cholesteryl esters in chylomicrons and extremely large VLDL	XXL_VLDL_CE	Lipoprotein subclasses	23485
Free cholesterol in chylomicrons and extremely large VLDL	XXL_VLDL_FC	Lipoprotein subclasses	23486
Triglycerides in chylomicrons and extremely large VLDL	XXL_VLDL_TG	Lipoprotein subclasses	23487
Concentration of very large VLDL particles	XL_VLDL_P	Lipoprotein subclasses	23488
Total lipids in very large VLDL	XL_VLDL_L	Lipoprotein subclasses	23489
Phospholipids in very large VLDL	XL_VLDL_PL	Lipoprotein subclasses	23490
Cholesterol in very large VLDL	XL_VLDL_C	Lipoprotein subclasses	23491
Cholesteryl esters in very large VLDL	XL_VLDL_CE	Lipoprotein subclasses	23492
Free cholesterol in very large VLDL	XL_VLDL_FC	Lipoprotein subclasses	23493
Triglycerides in very large VLDL	XL_VLDL_TG	Lipoprotein subclasses	23494
Concentration of large VLDL particles	L_VLDL_P	Lipoprotein subclasses	23495
Total lipids in large VLDL	L_VLDL_L	Lipoprotein subclasses	23496
Phospholipids in large VLDL	L_VLDL_PL	Lipoprotein subclasses	23497
Cholesterol in large VLDL	L_VLDL_C	Lipoprotein subclasses	23498
Cholesteryl esters in large VLDL	L_VLDL_CE	Lipoprotein subclasses	23499
Free cholesterol in large VLDL	L_VLDL_FC	Lipoprotein subclasses	23500
Triglycerides in large VLDL	L_VLDL_TG	Lipoprotein subclasses	23501
Concentration of medium VLDL particles	M_VLDL_P	Lipoprotein subclasses	23502
Total lipids in medium VLDL	M_VLDL_L	Lipoprotein subclasses	23503
Phospholipids in medium VLDL	M_VLDL_PL	Lipoprotein subclasses	23504
Cholesterol in medium VLDL	M_VLDL_C	Lipoprotein subclasses	23505
Cholesteryl esters in medium VLDL	M_VLDL_CE	Lipoprotein subclasses	23506
Free cholesterol in medium VLDL	M_VLDL_FC	Lipoprotein subclasses	23507
Triglycerides in medium VLDL	M_VLDL_TG	Lipoprotein subclasses	23508
Concentration of small VLDL particles	S_VLDL_P	Lipoprotein subclasses	23509
Total lipids in small VLDL	S_VLDL_L	Lipoprotein subclasses	23510
Phospholipids in small VLDL	S_VLDL_PL	Lipoprotein subclasses	23511
Cholesterol in small VLDL	S_VLDL_C	Lipoprotein subclasses	23512
Cholesteryl esters in small VLDL	S_VLDL_CE	Lipoprotein subclasses	23513
Free cholesterol in small VLDL	S_VLDL_FC	Lipoprotein subclasses	23514

Triglycerides in small VLDL	S_VLDL_TG	Lipoprotein subclasses	23515
Concentration of very small VLDL particles	XS_VLDL_P	Lipoprotein subclasses	23516
Total lipids in very small VLDL	XS_VLDL_L	Lipoprotein subclasses	23517
Phospholipids in very small VLDL	XS_VLDL_PL	Lipoprotein subclasses	23518
Cholesterol in very small VLDL	XS_VLDL_C	Lipoprotein subclasses	23519
Cholesteryl esters in very small VLDL	XS_VLDL_CE	Lipoprotein subclasses	23520
Free cholesterol in very small VLDL	XS_VLDL_FC	Lipoprotein subclasses	23521
Triglycerides in very small VLDL	XS_VLDL_TG	Lipoprotein subclasses	23522
Concentration of IDL particles	IDL_P	Lipoprotein subclasses	23523
Total lipids in IDL	IDL_L	Lipoprotein subclasses	23524
Phospholipids in IDL	IDL_PL	Lipoprotein subclasses	23525
Cholesterol in IDL	IDL_C	Lipoprotein subclasses	23526
Cholesteryl esters in IDL	IDL_CE	Lipoprotein subclasses	23527
Free cholesterol in IDL	IDL_FC	Lipoprotein subclasses	23528
Triglycerides in IDL	IDL_TG	Lipoprotein subclasses	23529
Concentration of large LDL particles	L_LDL_P	Lipoprotein subclasses	23530
Total lipids in large LDL	L_LDL_L	Lipoprotein subclasses	23531
Phospholipids in large LDL	L_LDL_PL	Lipoprotein subclasses	23532
Cholesterol in large LDL	L_LDL_C	Lipoprotein subclasses	23533
Cholesteryl esters in large LDL	L_LDL_CE	Lipoprotein subclasses	23534
Free cholesterol in large LDL	L_LDL_FC	Lipoprotein subclasses	23535
Triglycerides in large LDL	L_LDL_TG	Lipoprotein subclasses	23536
Concentration of medium LDL particles	M_LDL_P	Lipoprotein subclasses	23537
Total lipids in medium LDL	M_LDL_L	Lipoprotein subclasses	23538
Phospholipids in medium LDL	M_LDL_PL	Lipoprotein subclasses	23539
Cholesterol in medium LDL	M_LDL_C	Lipoprotein subclasses	23540
Cholesteryl esters in medium LDL	M_LDL_CE	Lipoprotein subclasses	23541
Free cholesterol in medium LDL	M_LDL_FC	Lipoprotein subclasses	23542
Triglycerides in medium LDL	M_LDL_TG	Lipoprotein subclasses	23543
Concentration of small LDL particles	S_LDL_P	Lipoprotein subclasses	23544
Total lipids in small LDL	S_LDL_L	Lipoprotein subclasses	23545
Phospholipids in small LDL	S_LDL_PL	Lipoprotein subclasses	23546
Cholesterol in small LDL	S_LDL_C	Lipoprotein subclasses	23547
Cholesteryl esters in small LDL	S_LDL_CE	Lipoprotein subclasses	23548
Free cholesterol in small LDL	S_LDL_FC	Lipoprotein subclasses	23549
Triglycerides in small LDL	S_LDL_TG	Lipoprotein subclasses	23550
Concentration of very large HDL particles	XL_HDL_P	Lipoprotein subclasses	23551
Total lipids in very large HDL	XL_HDL_L	Lipoprotein subclasses	23552
Phospholipids in very large HDL	XL_HDL_PL	Lipoprotein subclasses	23553
Cholesterol in very large HDL	XL_HDL_C	Lipoprotein subclasses	23554
Cholesteryl esters in very large HDL	XL_HDL_CE	Lipoprotein subclasses	23555
Free cholesterol in very large HDL	XL_HDL_FC	Lipoprotein subclasses	23556
Triglycerides in very large HDL	XL_HDL_TG	Lipoprotein subclasses	23557

Concentration of large HDL particles	L_HDL_P	Lipoprotein subclasses	23558
Total lipids in large HDL	L_HDL_L	Lipoprotein subclasses	23559
Phospholipids in large HDL	L_HDL_PL	Lipoprotein subclasses	23560
Cholesterol in large HDL	L_HDL_C	Lipoprotein subclasses	23561
Cholesteryl esters in large HDL	L_HDL_CE	Lipoprotein subclasses	23562
Free cholesterol in large HDL	L_HDL_FC	Lipoprotein subclasses	23563
Triglycerides in large HDL	L_HDL_TG	Lipoprotein subclasses	23564
Concentration of medium HDL particles	M_HDL_P	Lipoprotein subclasses	23565
Total lipids in medium HDL	M_HDL_L	Lipoprotein subclasses	23566
Phospholipids in medium HDL	M_HDL_PL	Lipoprotein subclasses	23567
Cholesterol in medium HDL	M_HDL_C	Lipoprotein subclasses	23568
Cholesteryl esters in medium HDL	M_HDL_CE	Lipoprotein subclasses	23569
Free cholesterol in medium HDL	M_HDL_FC	Lipoprotein subclasses	23570
Triglycerides in medium HDL	M_HDL_TG	Lipoprotein subclasses	23571
Concentration of small HDL particles	S_HDL_P	Lipoprotein subclasses	23572
Total lipids in small HDL	S_HDL_L	Lipoprotein subclasses	23573
Phospholipids in small HDL	S_HDL_PL	Lipoprotein subclasses	23574
Cholesterol in small HDL	S_HDL_C	Lipoprotein subclasses	23575
Cholesteryl esters in small HDL	S_HDL_CE	Lipoprotein subclasses	23576
Free cholesterol in small HDL	S_HDL_FC	Lipoprotein subclasses	23577
Triglycerides in small HDL	S_HDL_TG	Lipoprotein subclasses	23578
Phospholipids to total lipids ratio in chylomicrons and extremely large VLDL	XXL_VLDL_PL_pct	Relative lipoprotein lipid concentrations	23579
Cholesterol to total lipids ratio in chylomicrons and extremely large VLDL	XXL_VLDL_C_pct	Relative lipoprotein lipid concentrations	23580
Cholesteryl esters to total lipids ratio in chylomicrons and extremely large VLDL	XXL_VLDL_CE_pct	Relative lipoprotein lipid concentrations	23581
Free cholesterol to total lipids ratio in chylomicrons and extremely large VLDL	XXL_VLDL_FC_pct	Relative lipoprotein lipid concentrations	23582
Triglycerides to total lipids ratio in chylomicrons and extremely large VLDL	XXL_VLDL_TG_pct	Relative lipoprotein lipid concentrations	23583
Phospholipids to total lipids ratio in very large VLDL	XL_VLDL_PL_pct	Relative lipoprotein lipid concentrations	23584
Cholesterol to total lipids ratio in very large VLDL	XL_VLDL_C_pct	Relative lipoprotein lipid concentrations	23585
Cholesteryl esters to total lipids ratio in very large VLDL	XL_VLDL_CE_pct	Relative lipoprotein lipid concentrations	23586
Free cholesterol to total lipids ratio in very large VLDL	XL_VLDL_FC_pct	Relative lipoprotein lipid concentrations	23587
Triglycerides to total lipids ratio in very large VLDL	XL_VLDL_TG_pct	Relative lipoprotein lipid concentrations	23588
Phospholipids to total lipids ratio in large VLDL	L_VLDL_PL_pct	Relative lipoprotein lipid concentrations	23589
Cholesterol to total lipids ratio in large VLDL	L_VLDL_C_pct	Relative lipoprotein lipid concentrations	23590
Cholesteryl esters to total lipids ratio in large VLDL	L_VLDL_CE_pct	Relative lipoprotein lipid concentrations	23591
Free cholesterol to total lipids ratio in large VLDL	L_VLDL_FC_pct	Relative lipoprotein lipid concentrations	23592
Triglycerides to total lipids ratio in large VLDL	L_VLDL_TG_pct	Relative lipoprotein lipid concentrations	23593

Phospholipids to total lipids ratio in medium VLDL	M_VLDL_PL_pct	Relative lipoprotein lipid concentrations	23594
Cholesterol to total lipids ratio in medium VLDL	M_VLDL_C_pct	Relative lipoprotein lipid concentrations	23595
Cholesteryl esters to total lipids ratio in medium VLDL	M_VLDL_CE_pct	Relative lipoprotein lipid concentrations	23596
Free cholesterol to total lipids ratio in medium VLDL	M_VLDL_FC_pct	Relative lipoprotein lipid concentrations	23597
Triglycerides to total lipids ratio in medium VLDL	M_VLDL_TG_pct	Relative lipoprotein lipid concentrations	23598
Phospholipids to total lipids ratio in small VLDL	S_VLDL_PL_pct	Relative lipoprotein lipid concentrations	23599
Cholesterol to total lipids ratio in small VLDL	S_VLDL_C_pct	Relative lipoprotein lipid concentrations	23600
Cholesteryl esters to total lipids ratio in small VLDL	S_VLDL_CE_pct	Relative lipoprotein lipid concentrations	23601
Free cholesterol to total lipids ratio in small VLDL	S_VLDL_FC_pct	Relative lipoprotein lipid concentrations	23602
Triglycerides to total lipids ratio in small VLDL	S_VLDL_TG_pct	Relative lipoprotein lipid concentrations	23603
Phospholipids to total lipids ratio in very small VLDL	XS_VLDL_PL_pct	Relative lipoprotein lipid concentrations	23604
Cholesterol to total lipids ratio in very small VLDL	XS_VLDL_C_pct	Relative lipoprotein lipid concentrations	23605
Cholesteryl esters to total lipids ratio in very small VLDL	XS_VLDL_CE_pct	Relative lipoprotein lipid concentrations	23606
Free cholesterol to total lipids ratio in very small VLDL	XS_VLDL_FC_pct	Relative lipoprotein lipid concentrations	23607
Triglycerides to total lipids ratio in very small VLDL	XS_VLDL_TG_pct	Relative lipoprotein lipid concentrations	23608
Phospholipids to total lipids ratio in IDL	IDL_PL_pct	Relative lipoprotein lipid concentrations	23609
Cholesterol to total lipids ratio in IDL	IDL_C_pct	Relative lipoprotein lipid concentrations	23610
Cholesteryl esters to total lipids ratio in IDL	IDL_CE_pct	Relative lipoprotein lipid concentrations	23611
Free cholesterol to total lipids ratio in IDL	IDL_FC_pct	Relative lipoprotein lipid concentrations	23612
Triglycerides to total lipids ratio in IDL	IDL_TG_pct	Relative lipoprotein lipid concentrations	23613
Phospholipids to total lipids ratio in large LDL	L_LDL_PL_pct	Relative lipoprotein lipid concentrations	23614
Cholesterol to total lipids ratio in large LDL	L_LDL_C_pct	Relative lipoprotein lipid concentrations	23615
Cholesteryl esters to total lipids ratio in large LDL	L_LDL_CE_pct	Relative lipoprotein lipid concentrations	23616
Free cholesterol to total lipids ratio in large LDL	L_LDL_FC_pct	Relative lipoprotein lipid concentrations	23617
Triglycerides to total lipids ratio in large LDL	L_LDL_TG_pct	Relative lipoprotein lipid concentrations	23618
Phospholipids to total lipids ratio in medium LDL	M_LDL_PL_pct	Relative lipoprotein lipid concentrations	23619
Cholesterol to total lipids ratio in medium LDL	M_LDL_C_pct	Relative lipoprotein lipid concentrations	23620
Cholesteryl esters to total lipids ratio in medium LDL	M_LDL_CE_pct	Relative lipoprotein lipid concentrations	23621
Free cholesterol to total lipids ratio in medium LDL	M_LDL_FC_pct	Relative lipoprotein lipid concentrations	23622
Triglycerides to total lipids ratio in medium LDL	M_LDL_TG_pct	Relative lipoprotein lipid concentrations	23623
Phospholipids to total lipids ratio in small LDL	S_LDL_PL_pct	Relative lipoprotein lipid concentrations	23624
Cholesterol to total lipids ratio in small LDL	S_LDL_C_pct	Relative lipoprotein lipid concentrations	23625
Cholesteryl esters to total lipids ratio in small LDL	S_LDL_CE_pct	Relative lipoprotein lipid concentrations	23626
Free cholesterol to total lipids ratio in small LDL	S_LDL_FC_pct	Relative lipoprotein lipid concentrations	23627
Triglycerides to total lipids ratio in small LDL	S_LDL_TG_pct	Relative lipoprotein lipid concentrations	23628
Phospholipids to total lipids ratio in very large HDL	XL_HDL_PL_pct	Relative lipoprotein lipid concentrations	23629
Cholesterol to total lipids ratio in very large HDL	XL_HDL_C_pct	Relative lipoprotein lipid concentrations	23630
Cholesteryl esters to total lipids ratio in very large HDL	XL_HDL_CE_pct	Relative lipoprotein lipid concentrations	23631
Free cholesterol to total lipids ratio in very large HDL	XL_HDL_FC_pct	Relative lipoprotein lipid concentrations	23632

Triglycerides to total lipids ratio in very large HDL	XL_HDL_TG_pct	Relative lipoprotein lipid concentrations	23633
Phospholipids to total lipids ratio in large HDL	L_HDL_PL_pct	Relative lipoprotein lipid concentrations	23634
Cholesterol to total lipids ratio in large HDL	L_HDL_C_pct	Relative lipoprotein lipid concentrations	23635
Cholesteryl esters to total lipids ratio in large HDL	L_HDL_CE_pct	Relative lipoprotein lipid concentrations	23636
Free cholesterol to total lipids ratio in large HDL	L_HDL_FC_pct	Relative lipoprotein lipid concentrations	23637
Triglycerides to total lipids ratio in large HDL	L_HDL_TG_pct	Relative lipoprotein lipid concentrations	23638
Phospholipids to total lipids ratio in medium HDL	M_HDL_PL_pct	Relative lipoprotein lipid concentrations	23639
Cholesterol to total lipids ratio in medium HDL	M_HDL_C_pct	Relative lipoprotein lipid concentrations	23640
Cholesteryl esters to total lipids ratio in medium HDL	M_HDL_CE_pct	Relative lipoprotein lipid concentrations	23641
Free cholesterol to total lipids ratio in medium HDL	M_HDL_FC_pct	Relative lipoprotein lipid concentrations	23642
Triglycerides to total lipids ratio in medium HDL	M_HDL_TG_pct	Relative lipoprotein lipid concentrations	23643
Phospholipids to total lipids ratio in small HDL	S_HDL_PL_pct	Relative lipoprotein lipid concentrations	23644
Cholesterol to total lipids ratio in small HDL	S_HDL_C_pct	Relative lipoprotein lipid concentrations	23645
Cholesteryl esters to total lipids ratio in small HDL	S_HDL_CE_pct	Relative lipoprotein lipid concentrations	23646
Free cholesterol to total lipids ratio in small HDL	S_HDL_FC_pct	Relative lipoprotein lipid concentrations	23647
Triglycerides to total lipids ratio in small HDL	S_HDL_TG_pct	Relative lipoprotein lipid concentrations	23648
Glucose-lactate	GL	Glycolysis related metabolites	20280
Spectrometer-corrected alanine	SCA	Amino acids	20281

Table S3. Definitions of primary outcomes

**Entity**	**Abbr**	**New case identification**
Mental disorder	/	ICD-9: 290-319<br>ICD-10: F00-F99
Depressive disorder	/	ICD-9:311,296.1,296.3<br>ICD-10: F32, F33
Anxiety disorder	/	ICD-9: 300.0, 296.0, 296.2<br>ICD-10: F40, F41
Substance use disorder	SUD	ICD-9: 291,292,304,305<br>ICD-10: F10-F19

Table S4. Definitions of secondary outcomes

Category	UKB Data-Field	Code	Abbr
Subjective well-being	General happiness	20458	Unhappiness
	Happiness with own health	20459	Unhappiness with health
	Belief that own life is meaningful	20460	Meaningless feeling
Depression symptoms (Patient Health Questionnaire-9)	Recent thoughts of suicide or self-harm	20513	Suicidal ideation
	Trouble falling asleep, or sleeping too much	20517	Sleeping problems
	Recent changes in speed of moving or speaking	20518	Psychomotor changes
	Recent feelings of inadequacy	20507	Inadequacy feeling
	Recent feelings of tiredness or low energy	20519	Fatigue
	Recent feelings of depression	20510	Depression feeling
	Recent trouble concentrating on things	20508	Trouble concentrating
	Recent poor appetite or overeating	20511	Appetite changes
	Recent lack of interest or pleasure in doing things	20514	Anhedonia
Anxiety symptoms (Generalized Anxiety Disorder-7)	Recent inability to stop or control worrying	20509	Uncontrolled worry
	Recent restlessness	20516	Restlessness
	Recent trouble relaxing	20515	Trouble relaxing
	Recent easy annoyance or irritability	20505	Irritability
	Recent worrying too much about different things	20520	Excessive worry
	Recent feelings of foreboding	20512	Foreboding
	Recent feelings of nervousness or anxiety	20506	Anxiety feeling

Table S5. Definitions of baseline characteristics and healthy lifestyles

**Characteristics**	**Code**	**Definition**
Age	21022	This is a derived variable based on date of birth and date of attending an initial assessment center and refers to the age of the participant on the day they attended an Initial Assessment Center, truncated to whole year.
Sex	31	Acquired from central registry at recruitment, but in some cases updated by the participant. Hence this field may contain a mixture of the sex the National Health Service had recorded for the participant and self-reported sex.
Index of multiple deprivation (IMD)	26410<br>26427<br>26426	Domains for IMD calculation: crime score (England and Scotland), community safety score (Wales), education score (All), employment score (All), health score (All), housing score (All), income score (All), living environment score (England), access to services score (Scotland and Wales), physical environment score (Wales).<br>**Low**: <12.16;<br>**High**: ≥12.16.
Body mass index (BMI)	21001	BMI was calculated by dividing an individual's weight in kilograms by the square of their height in meters.
Waist-to-hip ratio (WHR)	48, 49	WHR was calculated through waist/hip measurement.<br>**Low**: <0.9 for men; <0.85 for women;<br>**High**: ≥0.9 for men; ≥0.85 for women.
Never smoking	20116	UK Biobank Touchscreen questionnaire at baseline;<br>This field summarized the current/past smoking status of the participant, divided into current, previous, and never smoker.<br>**Yes**: never smoking;<br>**No**: current and previous smoking.
Moderate drinking	20117	UK Biobank Touchscreen questionnaire at baseline;<br>No heavy alcohol intake was defined as the average daily intake ≤16 g of pure alcohol for both men and women.<br>**Yes**: average daily intake ≤16 g of pure alcohol (2 units of alcohol);<br>**No**: average daily intake >16 g of pure alcohol (2 units of alcohol).
Healthy sleep pattern	1180<br>1160<br>1200<br>1210<br>1220	Five aspects of healthy sleep behaviors: i) early chronotype; ii) sleep 7-8h/day; iii) never/rarely or sometimes insomnia; iv) no self-reported snoring; v) never/rarely or sometimes daytime dozing.<br>**Yes**: participants had ≥4 healthy components;<br>**No:** participants had <4 healthy components.
Regular physical activity	22035	This field indicated whether a participant met the 2017 UK physical activity guidelines.<br>**Yes**: participants had ≥150 minutes of moderate activity per week or ≥75 minutes of vigorous activity or equivalent combination;<br>**No**: participants had <150 minutes of moderate activity per week and <75 minutes of vigorous activity.
Healthy diet	1309, 1319<br>1289, 1299	Seven healthy diet components: i) Fruits: ≥ 3 servings/day; ii) Vegetables: ≥ 3 servings/day; iii) Fish: ≥2 servings/week; iv) Processed meats: ≤ 1 serving/week;

	1329, 1339<br>1349, 1369<br>1379, 1389<br>1438, 1448<br>1458, 1468	v) Unprocessed red meats: ≤ 1.5 servings/week; vi) Whole grains: ≥ 3 servings/day; vii) Refined grains: ≤1.5servings/day.<br>**Yes**: participants had ≥4 healthy diet components;<br>**No**: participants had <4 healthy diet components.

Table S6. New events, total person-years and incidence rates of mental disorders

Disease outcome	New Events, No.	Total person-years	Incidence Rate, per 100,000 person-years
Mental disorder	7594	333773	2275.2
Depressive disorder	865	376342	229.9
Anxiety disorder	892	376631	236.8
SUD	1300	372645	348.9

Abbreviations: SUD, Substance use disorder.

Table S7. Associations of ultra-processed food intake level and mental disorders by age and sex

Subgroup		Ultra-processed food intake level [a]			*P* trend	*P* for interaction
		Low	Moderate	High		
**Age**						
Mental disorder	<60	Ref.	1.05 (0.96, 1.14)	1.09 (1.00, 1.18)	0.044	0.464
	≥60	Ref.	1.01 (0.94, 1.09)	1.04 (0.96, 1.13)	0.310	
Depressive disorder	<60	Ref.	1.11 (0.86, 1.42)	1.55 (1.23, 1.94)	<0.001	0.077
	≥60	Ref.	1.13 (0.89, 1.44)	1.14 (0.89, 1.47)	0.288	
Anxiety disorder	<60	Ref.	1.21 (0.95, 1.54)	1.34 (1.06, 1.69)	0.014	0.806
	≥60	Ref.	1.13 (0.89, 1.42)	1.29 (1.02, 1.63)	0.036	
SUD	<60	Ref.	1.10 (0.91, 1.32)	1.20 (1.01, 1.44)	0.041	0.009
	≥60	Ref.	0.97 (0.80, 1.18)	0.84 (0.68, 1.03)	0.105	
**Sex**						
Mental disorder	Male	Ref.	1.04 (0.96, 1.13)	1.06 (0.97, 1.15)	0.194	0.513
	Female	Ref.	1.02 (0.94, 1.10)	1.10 (1.01, 1.18)	0.020	
Depressive disorder	Male	Ref.	0.99 (0.75, 1.31)	1.26 (0.97, 1.63)	0.071	0.485
	Female	Ref.	1.21 (0.97, 1.51)	1.42 (1.14, 1.76)	0.002	
Anxiety disorder	Male	Ref.	1.30 (0.98, 1.72)	1.27 (0.96, 1.68)	0.114	0.694
	Female	Ref.	1.09 (0.89, 1.35)	1.36 (1.11, 1.66)	0.003	
SUD	Male	Ref.	0.99 (0.83, 1.18)	0.99 (0.83, 1.17)	0.877	0.445
	Female	Ref.	1.10 (0.89, 1.36)	1.10 (0.89, 1.36)	0.399	

Abbreviations: SUD, Substance use disorder. **a** Participants' ultra-processed food intake was categorized into tertiles based on the distribution and classified as low, moderate, and high levels, with the low level serving as the reference group.

Table S8. Associations of ultra-processed food intake and mental disorders by age and sex

Subgroup		HR (95% CI) [a]	*P* value	*P* for interaction
**Age**				
Mental disorder	<60	1.05 (1.02, 1.07)	0.001	0.128
	≥60	1.01 (0.98, 1.05)	0.394	
Depressive disorder	<60	1.19 (1.12, 1.26)	<0.001	0.031
	≥60	1.05 (0.96, 1.15)	0.261	
Anxiety disorder	<60	1.12 (1.05, 1.20)	0.001	0.651
	≥60	1.09 (1.00, 1.19)	0.047	
SUD	<60	1.13 (1.07, 1.19)	<0.001	<0.001
	≥60	0.94 (0.86, 1.02)	0.124	
**Sex**				
Mental disorder	Male	1.03 (1.00, 1.06)	0.058	0.319
	Female	1.05 (1.02, 1.08)	0.001	
Depressive disorder	Male	1.12 (1.03, 1.22)	0.008	0.597
	Female	1.15 (1.08, 1.23)	<0.001	
Anxiety disorder	Male	1.05 (0.95, 1.15)	0.340	0.090
	Female	1.15 (1.08, 1.23)	<0.001	
SUD	Male	1.03 (0.97, 1.09)	0.363	0.166
	Female	1.10 (1.02, 1.18)	0.009	

Abbreviations: SUD, Substance use disorder. **a** Participants' ultra-processed food intake was analyzed as a continuous variable with per 10% increment.

Table S9. Associations of metabolic signature score level and mental disorders by age and sex

Subgroup		Metabolic signature score level [a]			*P* trend	*P* for interaction
		Low	Moderate	High		
**Age**						
Mental disorder	<60	Ref.	1.04 (0.95, 1.13)	1.21 (1.12, 1.32)	<0.001	0.188
	≥60	Ref.	1.09 (1.01, 1.18)	1.13 (1.04, 1.22)	0.004	
Depressive disorder	<60	Ref.	1.15 (0.89, 1.48)	1.56 (1.23, 1.97)	<0.001	0.036
	≥60	Ref.	1.05 (0.82, 1.34)	1.09 (0.85, 1.40)	0.512	
Anxiety disorder	<60	Ref.	1.24 (0.97, 1.60)	1.64 (1.29, 2.08)	<0.001	0.021
	≥60	Ref.	1.09 (0.87, 1.38)	1.11 (0.87, 1.42)	0.385	
SUD	<60	Ref.	1.10 (0.89, 1.35)	1.80 (1.49, 2.18)	<0.001	0.061
	≥60	Ref.	1.01 (0.81, 1.26)	1.39 (1.13, 1.71)	0.001	
**Sex**						
Mental disorder	Male	Ref.	1.04 (0.95, 1.14)	1.13 (1.03, 1.23)	0.004	0.146
	Female	Ref.	1.08 (1.01, 1.17)	1.23 (1.13, 1.33)	<0.001	
Depressive disorder	Male	Ref.	1.13 (0.81, 1.57)	1.46 (1.08, 1.96)	0.006	0.385
	Female	Ref.	1.09 (0.88, 1.35)	1.24 (0.99, 1.54)	0.057	
Anxiety disorder	Male	Ref.	1.01 (0.74, 1.38)	1.10 (0.82, 1.47)	0.471	0.070
	Female	Ref.	1.22 (1.00, 1.50)	1.52 (1.23, 1.88)	<0.001	
SUD	Male	Ref.	0.98 (0.80, 1.20)	1.35 (1.12, 1.63)	<0.001	0.004
	Female	Ref.	1.10 (0.88, 1.38)	2.01 (1.63, 2.48)	<0.001	

Abbreviations: SUD, Substance use disorder. **a** Participants' metabolic signature was categorized into tertiles based on the distribution and classified as low, moderate, and high levels, with the low level serving as the reference group.

Table S10. Associations of metabolic signature score and mental disorders by age and sex

Subgroup		HR (95% CI) [a]	*P* value	*P* for interaction
**Age**				
Mental disorder	<60	1.09 (1.05, 1.13)	<0.001	0.129
	≥60	1.05 (1.02, 1.09)	0.002	
Depressive disorder	<60	1.23 (1.12, 1.35)	<0.001	0.042
	≥60	1.07 (0.97, 1.19)	0.169	
Anxiety disorder	<60	1.24 (1.13, 1.36)	<0.001	0.006
	≥60	1.04 (0.94, 1.15)	0.486	
SUD	<60	1.32 (1.24, 1.41)	<0.001	0.015
	≥60	1.17 (1.07, 1.26)	<0.001	
**Sex**				
Mental disorder	Male	1.07 (1.03, 1.10)	<0.001	0.347
	Female	1.09 (1.05, 1.13)	<0.001	
Depressive disorder	Male	1.23 (1.11, 1.36)	<0.001	0.115
	Female	1.10 (1.01, 1.21)	0.035	
Anxiety disorder	Male	1.03 (0.93, 1.15)	0.551	0.014
	Female	1.23 (1.12, 1.34)	<0.001	
SUD	Male	1.19 (1.11, 1.27)	<0.001	0.005
	Female	1.38 (1.27, 1.51)	<0.001	

Abbreviations: SUD, Substance use disorder; SD, standard deviation. **a** Participants' metabolic signature was analyzed as a continuous variable with per SD increment.

Table S11. Associations of ultra-processed food intake and mental health symptoms by age

Symptom	<60		≥60		*P* for interaction
	OR (95% CI)	*P* FDR	OR (95% CI)	*P* FDR	
Unhappiness	1.07 (0.98, 1.16)	0.134	0.94 (0.78, 1.12)	0.627	0.767
Unhappiness with health	1.08 (1.01, 1.14)	0.035	1.09 (0.99, 1.19)	0.239	0.972
Meaningless feeling	1.11 (1.03, 1.19)	0.011	1.10 (0.97, 1.24)	0.258	0.972
Suicidal ideation	1.14 (1.03, 1.25)	0.012	1.14 (0.97, 1.33)	0.258	0.972
Sleeping problems	1.09 (1.04, 1.13)	0.001	1.03 (0.97, 1.10)	0.441	0.767
Psychomotor changes	1.16 (1.06, 1.26)	0.002	1.09 (0.93, 1.26)	0.437	0.869
Inadequacy feeling	1.08 (1.03, 1.14)	0.004	1.01 (0.93, 1.10)	0.873	0.767
Fatigue	1.05 (1.00, 1.10)	0.037	1.01 (0.95, 1.07)	0.873	0.767
Depression feeling	1.07 (1.02, 1.12)	0.011	1.00 (0.92, 1.08)	0.933	0.767
Trouble concentrating	1.12 (1.06, 1.18)	<0.001	1.01 (0.93, 1.10)	0.873	0.767
Appetite changes	1.11 (1.05, 1.16)	0.001	1.11 (1.02, 1.21)	0.135	0.972
Anhedonia	1.10 (1.04, 1.16)	0.001	1.05 (0.96, 1.14)	0.437	0.823
Uncontrolled worry	1.06 (1.00, 1.11)	0.037	1.09 (1.01, 1.17)	0.135	0.869
Restlessness	1.12 (1.06, 1.19)	0.001	1.07 (0.96, 1.18)	0.379	0.829
Trouble relaxing	1.09 (1.04, 1.14)	0.001	1.08 (1.01, 1.16)	0.135	0.972
Irritability	1.05 (1.00, 1.10)	0.040	1.03 (0.96, 1.10)	0.600	0.934
Excessive worry	1.08 (1.03, 1.13)	0.002	1.06 (0.99, 1.13)	0.249	0.934
Foreboding	1.09 (1.03, 1.15)	0.004	1.08 (0.99, 1.17)	0.249	0.972
Anxiety feeling	1.05 (1.00, 1.10)	0.037	1.10 (1.03, 1.18)	0.129	0.767

Abbreviations: FDR, false discovery rate.

Table S12. Associations of ultra-processed food intake and mental health symptoms by sex

Symptom	Male		Female		*P* for interaction
	**OR (95% CI)**	***P* FDR**	**OR (95% CI)**	***P* FDR**	
Unhappiness	1.06 (0.95, 1.18)	0.293	0.97 (0.87, 1.09)	0.625	0.789
Unhappiness with health	1.11 (1.03, 1.20)	0.025	1.03 (0.96, 1.11)	0.466	0.789
Meaningless feeling	1.13 (1.03, 1.23)	0.03	1.05 (0.96, 1.15)	0.342	0.789
Suicidal ideation	1.14 (1.01, 1.28)	0.049	1.10 (0.97, 1.23)	0.244	0.789
Sleeping problems	1.05 (1.00, 1.11)	0.092	1.07 (1.02, 1.12)	0.067	0.789
Psychomotor changes	1.09 (0.98, 1.22)	0.131	1.16 (1.05, 1.27)	0.067	0.789
Inadequacy feeling	1.07 (1.00, 1.14)	0.075	1.03 (0.97, 1.09)	0.462	0.789
Fatigue	1.01 (0.96, 1.06)	0.712	1.03 (0.98, 1.08)	0.331	0.789
Depression feeling	1.06 (0.99, 1.13)	0.092	1.02 (0.96, 1.08)	0.534	0.789
Trouble concentrating	1.07 (1.00, 1.14)	0.067	1.08 (1.02, 1.14)	0.067	0.915
Appetite changes	1.11 (1.03, 1.19)	0.025	1.07 (1.01, 1.14)	0.067	0.789
Anhedonia	1.07 (1.00, 1.14)	0.067	1.07 (1.01, 1.13)	0.096	0.961
Uncontrolled worry	1.08 (1.01, 1.15)	0.049	1.04 (0.99, 1.09)	0.248	0.789
Restlessness	1.11 (1.02, 1.20)	0.032	1.09 (1.01, 1.16)	0.067	0.823
Trouble relaxing	1.10 (1.04, 1.16)	0.015	1.05 (1.00, 1.10)	0.162	0.789
Irritability	1.03 (0.97, 1.09)	0.302	1.02 (0.97, 1.08)	0.466	0.912
Excessive worry	1.10 (1.04, 1.16)	0.015	1.04 (0.99, 1.09)	0.248	0.789
Foreboding	1.10 (1.02, 1.18)	0.03	1.05 (0.99, 1.12)	0.206	0.789
Anxiety feeling	1.07 (1.01, 1.14)	0.049	1.04 (0.99, 1.09)	0.244	0.789

Abbreviations: FDR, false discovery rate.

Table S13. Associations of metabolic signature score and mental health symptoms by age

Symptom	<60		≥60		*P* for interaction
	OR (95% CI)	*P* FDR	OR (95% CI)	*P* FDR	
Unhappiness	1.14 (1.02, 1.26)	0.051	0.98 (0.83, 1.16)	0.952	0.173
Unhappiness with health	1.12 (1.04, 1.22)	0.015	0.90 (0.82, 0.99)	0.099	0.002
Meaningless feeling	1.11 (1.01, 1.22)	0.059	1.00 (0.88, 1.14)	0.952	0.219
Suicidal ideation	1.23 (1.08, 1.39)	0.008	0.99 (0.83, 1.18)	0.952	0.067
Sleeping problems	1.05 (1.00, 1.11)	0.111	0.96 (0.90, 1.02)	0.297	0.035
Psychomotor changes	1.08 (0.97, 1.21)	0.217	0.97 (0.83, 1.14)	0.928	0.275
Inadequacy feeling	1.10 (1.03, 1.17)	0.015	0.94 (0.86, 1.03)	0.297	0.012
Fatigue	1.08 (1.02, 1.13)	0.015	1.00 (0.94, 1.06)	0.952	0.087
Depression feeling	1.11 (1.04, 1.17)	0.008	0.91 (0.84, 0.99)	0.097	0.002
Trouble concentrating	1.05 (0.99, 1.12)	0.189	0.96 (0.88, 1.05)	0.522	0.112
Appetite changes	1.05 (0.98, 1.12)	0.217	1.10 (1.00, 1.21)	0.099	0.354
Anhedonia	1.13 (1.06, 1.21)	0.003	0.94 (0.86, 1.03)	0.297	0.003
Uncontrolled worry	1.04 (0.98, 1.11)	0.217	0.91 (0.85, 0.99)	0.097	0.012
Restlessness	1.05 (0.98, 1.14)	0.22	0.96 (0.86, 1.07)	0.603	0.173
Trouble relaxing	1.05 (1.00, 1.11)	0.121	0.91 (0.85, 0.98)	0.097	0.007
Irritability	1.02 (0.97, 1.08)	0.406	0.92 (0.86, 0.99)	0.097	0.035
Excessive worry	1.04 (0.98, 1.09)	0.221	0.89 (0.83, 0.95)	0.014	0.002
Foreboding	1.08 (1.01, 1.16)	0.051	0.93 (0.85, 1.02)	0.227	0.012
Anxiety feeling	1.00 (0.94, 1.05)	0.896	0.91 (0.85, 0.98)	0.097	0.073

Abbreviations: FDR, false discovery rate.

Table S14. Associations of metabolic signature score and mental health symptoms by sex

Symptom	Male		Female		*P* for interaction
	OR (95% CI)	*P* FDR	OR (95% CI)	*P* FDR	
Unhappiness	1.07 (0.94, 1.22)	0.531	1.06 (0.93, 1.21)	0.839	0.923
Unhappiness with health	1.04 (0.95, 1.13)	0.615	1.00 (0.92, 1.10)	0.954	0.923
Meaningless feeling	1.03 (0.92, 1.15)	0.754	1.09 (0.98, 1.21)	0.512	0.830
Suicidal ideation	1.15 (0.99, 1.32)	0.313	1.11 (0.96, 1.28)	0.636	0.923
Sleeping problems	1.02 (0.97, 1.09)	0.615	0.99 (0.94, 1.04)	0.950	0.830
Psychomotor changes	1.04 (0.91, 1.19)	0.705	1.03 (0.91, 1.17)	0.950	0.923
Inadequacy feeling	1.08 (1.00, 1.16)	0.313	0.99 (0.93, 1.06)	0.950	0.525
Fatigue	1.07 (1.01, 1.13)	0.313	1.01 (0.95, 1.06)	0.950	0.557
Depression feeling	1.05 (0.97, 1.12)	0.531	1.01 (0.94, 1.08)	0.950	0.830
Trouble concentrating	1.02 (0.94, 1.10)	0.754	1.00 (0.93, 1.07)	0.954	0.923
Appetite changes	1.09 (0.99, 1.18)	0.313	1.02 (0.95, 1.09)	0.950	0.787
Anhedonia	1.06 (0.99, 1.15)	0.432	1.04 (0.97, 1.12)	0.839	0.923
Uncontrolled worry	0.99 (0.92, 1.07)	0.792	0.98 (0.92, 1.05)	0.950	0.923
Restlessness	1.07 (0.97, 1.18)	0.531	0.97 (0.89, 1.05)	0.894	0.525
Trouble relaxing	1.04 (0.97, 1.11)	0.531	0.95 (0.89, 1.01)	0.503	0.525
Irritability	0.98 (0.91, 1.04)	0.615	0.97 (0.91, 1.03)	0.839	0.923
Excessive worry	1.01 (0.95, 1.08)	0.756	0.94 (0.89, 0.99)	0.495	0.525
Foreboding	1.05 (0.96, 1.14)	0.531	0.99 (0.93, 1.07)	0.950	0.830
Anxiety feeling	0.97 (0.91, 1.04)	0.615	0.94 (0.89, 1.00)	0.495	0.923

Abbreviations: FDR, false discovery rate.

Table S15. Sensitivity analysis of the main associations

Sensitivity analysis [a]	Variable	Continuous [b]		Categorical [c]			
		HR (95% CI)	*P* value	Low	Moderate	High	*P* trend
**Sensitivity analysis 1**							
Mental disorder	UPF	1.05 (1.02, 1.07)	<0.001	Ref.	1.00 (0.94, 1.05)	1.05 (0.99, 1.11)	0.106
	Signature	1.06 (1.04, 1.09)	<0.001	Ref.	1.08 (1.02, 1.14)	1.16 (1.10, 1.23)	<0.001
Depressive disorder	UPF	1.14 (1.08, 1.21)	<0.001	Ref.	1.03 (0.87, 1.23)	1.23 (1.04, 1.45)	0.013
	Signature	1.15 (1.08, 1.24)	<0.001	Ref.	1.05 (0.88, 1.25)	1.31 (1.10, 1.56)	0.002
Anxiety disorder	UPF	1.13 (1.06, 1.20)	<0.001	Ref.	1.05 (0.89, 1.24)	1.28 (1.09, 1.50)	0.003
	Signature	1.17 (1.09, 1.25)	<0.001	Ref.	1.25 (1.05, 1.49)	1.57 (1.32, 1.88)	<0.001
SUD	UPF	1.06 (1.01, 1.12)	0.022	Ref.	1.02 (0.89, 1.17)	1.06 (0.93, 1.21)	0.412
	Signature	1.21 (1.15, 1.28)	<0.001	Ref.	1.26 (1.08, 1.47)	1.65 (1.42, 1.91)	<0.001
**Sensitivity analysis 2**							
Mental disorder	UPF	1.04 (1.01, 1.06)	0.001	Ref.	1.02 (0.96, 1.08)	1.07 (1.01, 1.13)	0.027
	Signature	1.07 (1.05, 1.10)	<0.001	Ref.	1.07 (1.01, 1.14)	1.17 (1.10, 1.25)	<0.001
Depressive disorder	UPF	1.14 (1.08, 1.20)	<0.001	Ref.	1.10 (0.92, 1.31)	1.33 (1.12, 1.59)	0.001
	Signature	1.16 (1.08, 1.25)	<0.001	Ref.	1.07 (0.89, 1.28)	1.29 (1.07, 1.55)	0.006
Anxiety disorder	UPF	1.13 (1.07, 1.19)	<0.001	Ref.	1.17 (0.99, 1.39)	1.34 (1.13, 1.59)	0.001
	Signature	1.16 (1.07, 1.24)	<0.001	Ref.	1.20 (1.01, 1.43)	1.41 (1.17, 1.68)	<0.001
SUD	UPF	1.05 (0.99, 1.10)	0.081	Ref.	1.01 (0.88, 1.16)	1.01 (0.87, 1.16)	0.937
	Signature	1.25 (1.18, 1.32)	<0.001	Ref.	1.06 (0.91, 1.24)	1.59 (1.37, 1.85)	<0.001
**Sensitivity analysis 3**							
Mental disorder	UPF	1.04 (1.02, 1.07)	<0.001	Ref.	1.05 (0.99, 1.12)	1.10 (1.03, 1.17)	0.003
	Signature	1.08 (1.05, 1.11)	<0.001	Ref.	1.06 (1.00, 1.13)	1.18 (1.11, 1.26)	<0.001
Depressive disorder	UPF	1.14 (1.08, 1.20)	<0.001	Ref.	1.13 (0.93, 1.37)	1.39 (1.16, 1.67)	<0.001
	Signature	1.18 (1.10, 1.28)	<0.001	Ref.	1.08 (0.89, 1.31)	1.34 (1.11, 1.62)	0.002
Anxiety disorder	UPF	1.11 (1.05, 1.18)	0.001	Ref.	1.14 (0.95, 1.37)	1.30 (1.08, 1.56)	0.005
	Signature	1.16 (1.07, 1.25)	<0.001	Ref.	1.14 (0.95, 1.38)	1.37 (1.13, 1.66)	0.001
SUD	UPF	1.05 (1.00, 1.10)	0.070	Ref.	1.02 (0.89, 1.18)	0.99 (0.86, 1.15)	0.945
	Signature	1.24 (1.17, 1.32)	<0.001	Ref.	1.05 (0.90, 1.24)	1.56 (1.34, 1.81)	<0.001
**Sensitivity analysis 4**							
Mental disorder	UPF	1.04 (1.02, 1.07)	<0.001	Ref.	1.02 (0.96, 1.08)	1.08 (1.01, 1.14)	0.019
	Signature	1.08 (1.05, 1.11)	<0.001	Ref.	1.09 (1.02, 1.16)	1.19 (1.11, 1.27)	<0.001
Depressive disorder	UPF	1.15 (1.09, 1.22)	<0.001	Ref.	1.10 (0.91, 1.33)	1.34 (1.12, 1.61)	0.001
	Signature	1.15 (1.07, 1.25)	<0.001	Ref.	1.13 (0.93, 1.37)	1.33 (1.10, 1.61)	0.004
Anxiety disorder	UPF	1.12 (1.06, 1.19)	<0.001	Ref.	1.14 (0.95, 1.36)	1.30 (1.09, 1.55)	0.004
	Signature	1.14 (1.06, 1.23)	0.001	Ref.	1.20 (1.00, 1.43)	1.33 (1.11, 1.61)	0.003
SUD	UPF	1.07 (1.01, 1.12)	0.013	Ref.	1.04 (0.90, 1.20)	1.04 (0.90, 1.21)	0.571
	Signature	1.25 (1.18, 1.32)	<0.001	Ref.	1.05 (0.89, 1.24)	1.60 (1.37, 1.86)	<0.001
**Sensitivity analysis 5**							
Mental disorder	UPF	1.04 (1.02, 1.07)	<0.001	Ref.	1.02 (0.96, 1.08)	1.09 (1.02, 1.15)	0.006
	Signature	1.09 (1.06, 1.12)	<0.001	Ref.	1.08 (1.01, 1.15)	1.21 (1.14, 1.29)	<0.001
Depressive disorder	UPF	1.13 (1.06, 1.19)	<0.001	Ref.	1.07 (0.89, 1.29)	1.30 (1.09, 1.56)	0.003
	Signature	1.16 (1.07, 1.25)	<0.001	Ref.	1.06 (0.88, 1.29)	1.32 (1.09, 1.59)	0.003

Anxiety disorder	UPF	1.12 (1.05, 1.19)	<0.001	Ref.	1.12 (0.94, 1.34)	1.29 (1.08, 1.54)	0.004
	Signature	1.14 (1.06, 1.23)	0.001	Ref.	1.12 (0.93, 1.34)	1.38 (1.15, 1.67)	0.001
SUD	UPF	1.07 (1.01, 1.12)	0.013	Ref.	1.05 (0.91, 1.21)	1.04 (0.90, 1.20)	0.609
	Signature	1.27 (1.20, 1.35)	<0.001	Ref.	1.00 (0.85, 1.17)	1.63 (1.41, 1.90)	<0.001
**Sensitivity analysis 6**							
Mental disorder	UPF	1.04 (1.02, 1.06)	<0.001	Ref.	1.02 (0.97, 1.08)	1.07 (1.01, 1.13)	0.017
	Signature	1.07 (1.05, 1.10)	<0.001	Ref.	1.07 (1.01, 1.13)	1.17 (1.10, 1.25)	<0.001
Depressive disorder	UPF	1.14 (1.08, 1.20)	<0.001	Ref.	1.11 (0.94, 1.33)	1.34 (1.14, 1.59)	<0.001
	Signature	1.15 (1.07, 1.24)	<0.001	Ref.	1.1 (0.92, 1.31)	1.31 (1.10, 1.56)	0.002
Anxiety disorder	UPF	1.11 (1.05, 1.18)	<0.001	Ref.	1.16 (0.98, 1.37)	1.32 (1.11, 1.55)	0.001
	Signature	1.14 (1.06, 1.22)	<0.001	Ref.	1.16 (0.98, 1.38)	1.35 (1.14, 1.62)	0.001
SUD	UPF	1.06 (1.01, 1.11)	0.029	Ref.	1.03 (0.90, 1.18)	1.02 (0.89, 1.17)	0.740
	Signature	1.25 (1.18, 1.32)	<0.001	Ref.	1.05 (0.90, 1.22)	1.59 (1.37, 1.84)	<0.001

Abbreviations: SUD, Substance use disorder; HR, hazard ratio; CI, confidence interval. **a** Multivariable model adjusted for age, sex, IMD, BMI, WHR, healthy lifestyle factors and prevalent diseases. Sensitivity analysis 1: using the absolute amount of UPF intake (grams/day) instead of the proportion; sensitivity analysis 2: excluding non-white participants; sensitivity analysis 3: advancing the end date of follow-up to December 31, 2019; sensitivity analysis 4: excluding those diagnosed with any targeted events in the first two years of follow-up.; sensitivity analysis 5: controlling for additional environmental factors (proximity to roadways, noise pollution, nitrogen oxide, and fine particulate matter); sensitivity analysis 6: using Fine-Gray subdistribution hazard models to account for competing risk events. **b** Ultra-processed food intake was analyzed as a continuous variable with per 10% increment, and metabolic signature was analyzed as a continuous variable with per SD increment. **c** Participants' ultra-processed food intake and related metabolic signature were categorized into tertiles based on the distribution and classified as low, moderate, and high levels, with the low level serving as the reference group.

Table S16. Baseline characteristics of participants grouped by completion of the mental health questionnaire

Characteristics	All participants	Completion of the mental health questionnaire [a]		*P* value [b]
		No (n=17,888)	Yes (n=12,171)	
Age	56.5(8.05)	56.5(8.25)	56.5(7.74)	0.994
Sex				<0.001
Female	15956(53.1%)	9223(51.6%)	6733(55.3%)	
Male	14103(46.9%)	8665(48.4%)	5438(44.7%)	
IMD c				<0.001
Low	15041(50.0%)	8384(46.9%)	6657(54.7%)	
High	15018(50.0%)	9504(53.1%)	5514(45.3%)	
BMI	27.1(4.65)	27.4(4.72)	26.6(4.50)	<0.001
WHR [d]				<0.001
Low	15578(51.8%)	8740(48.9%)	6838(56.2%)	
High	14481(48.2%)	9148(51.1%)	5333(43.8%)	
Healthy lifestyle				
Never smoking [e]	17485(58.2%)	10307(57.6%)	7178(59.0%)	0.020
Moderate drinking [f]	20277(67.5%)	12150(67.9%)	8127(66.8%)	0.038
Regular physical activity [g]	16717(55.6%)	9706(54.3%)	7011(57.6%)	<0.001
Healthy sleep pattern [h]	16930(56.3%)	10152(56.8%)	6778(55.7%)	0.070
Healthy diet [i]	11708(39.0%)	6721(37.6%)	4987(41.0%)	<0.001
Prevalent disease				
Hypertension	7943(26.4%)	5143(28.8%)	2800(23.0%)	<0.001
Diabetes	606(2.02%)	413(2.31%)	193(1.59%)	<0.001
Coronary artery disease	1378(4.58%)	967(5.41%)	411(3.38%)	<0.001

Abbreviations: IMD, index of multiple deprivation; BMI, body mass index; WHR, waist-to-hip ratio. **a** After exclusions, a total of 30,059 participants were included in the final analysis, of whom 12,171 had completed the online mental health questionnaire (response rate: 42.3%). **b** Group comparisons were conducted using analysis of variance (ANOVA) or the chi-square (χ^2) test, as appropriate. **c** IMD was calculated through indicators across several different domains, including crime score, community safety score, education score, employment score, health score, housing score, income score, living environment score, access to services score, and physical environment score, and was categorized as low (<12.16) and high (≥12.16). **d** The WHR was calculated as waist circumference (cm) divided by hip circumference (cm) and classified as low (<0.9 for men and <0.85 for women) or high (≥0.9 for men and ≥0.85 for women). **e** Never smoking was defined as the absence of both past and current smoking at baseline. **f** Moderate drinking was defined as an average daily intake of ≤16 g of pure alcohol (equivalent to ≤2 units) for both men and women. **g** Regular physical activity was defined as engagement in at least 150 minutes of moderate-intensity activity per week, at least 75 minutes of vigorous-intensity activity per week, or an equivalent combination of both. h A healthy sleep pattern was assessed based on five dimensions of sleep behavior: early chronotype, sleep duration of 7–8 hours per day, absence of frequent insomnia (never/rarely or sometimes), no self-reported snoring, and absence of excessive daytime sleepiness (never/rarely or sometimes). Participants meeting at least four of these five criteria were classified as having a healthy sleep pattern. **i** A healthy diet was defined as the consumption of at least four out of seven key food groups prioritized for cardiometabolic health. The specific criteria for each component were as follows: ≥3 servings/day of fruits, ≥3 servings/day of vegetables, ≥2 servings/week of fish, ≤1 serving/week of processed meats, ≤1.5 servings/week of unprocessed red meats, ≥3 servings/day of whole grains, and ≤1.5 servings/day of refined grains.
